# Machine learning methods to predict 30-day hospital readmission outcome among US adults with pneumonia: analysis of the national readmission database

**DOI:** 10.1186/s12911-022-01995-3

**Published:** 2022-11-09

**Authors:** Yinan Huang, Ashna Talwar, Ying Lin, Rajender R. Aparasu

**Affiliations:** 1 Department of Pharmaceutical Health Outcomes and Policy, College of Pharmacy, University of Houston, 4849 Calhoun Road, Health and Sciences Bldg 2, Houston, TX 77204 USA; 2 Department of Industrial Engineering, Cullen College of Engineering, University of Houston, Houston, TX USA

**Keywords:** Machine learning, Rule-based learning, Random forest, XGBoost, Hospital readmission

## Abstract

**Background:**

Hospital readmissions for pneumonia are a growing concern in the US, with significant consequences for costs and quality of care. This study developed the rule-based model and other machine learning (ML) models to predict 30-day readmission risk in patients with pneumonia and compared model performance.

**Methods:**

This population-based study involved patients aged ≥ 18 years hospitalized with pneumonia from January 1, 2016, through November 30, 2016, using the Healthcare Cost and Utilization Project-National Readmission Database (HCUP-NRD). Rule-based algorithms and other ML algorithms, specifically decision trees, random forest, extreme gradient descent boosting (XGBoost), and Least Absolute Shrinkage and Selection Operator (LASSO), were used to model all-cause readmissions 30 days post-discharge from index pneumonia hospitalization. A total of 61 clinically relevant variables were included for ML model development. Models were trained on randomly partitioned 50% of the data and evaluated using the remaining dataset. Model hyperparameters were tuned using the ten-fold cross-validation on the resampled training dataset. The area under the receiver operating curves (AUROC) and area under precision-recall curves (AUPRC) were calculated for the testing set to evaluate the model performance.

**Results:**

Of the 372,293 patients with an index hospital hospitalization for pneumonia, 48,280 (12.97%) were readmitted within 30 days. Judged by AUROC in the testing data, rule-based model (0.6591) significantly outperformed decision tree (0.5783, *p* value < 0.001), random forest (0.6509, *p* value < 0.01) and LASSO (0.6087, *p* value < 0.001), but was less superior than XGBoost (0.6606, *p* value = 0.015). The AUPRC of the rule-based model in the testing data (0.2146) was higher than the decision tree (0.1560), random forest (0.2052), and LASSO (0.2042), but was similar to XGBoost (0.2147). The top risk-predictive rules captured by the rule-based algorithm were comorbidities, illness severity, disposition locations, payer type, age, and length of stay. These predictive risk factors were also identified by other ML models with high variable importance.

**Conclusion:**

The performance of machine learning models for predicting readmission in pneumonia patients varied. The XGboost was better than the rule-based model based on the AUROC. However, important risk factors for predicting readmission remained consistent across ML models.

**Supplementary Information:**

The online version contains supplementary material available at 10.1186/s12911-022-01995-3.

## Background

Pneumonia is one of the leading medical conditions causing hospitalizations in the US [1, 2]. With problems of the aging population and the increased antibiotic resistance, the hospitalizations for pneumonia in the US have increased dramatically in recent years, particularly affecting the elderly and those with underlying chronic conditions [3–5]. Hospital readmissions among patients hospitalized for pneumonia are frequent, with one in five patients requiring readmission [6, 7]. In alignment with the strategy of improving the quality of care through value-based payment, hospital readmission has become a strong quality metric linked to hospital reimbursement in the US [8]. In the Hospital Readmissions Reduction Program (HRRP) launched by the Affordable Care Act (ACA) of 2010, the Center for Medicare & Medicaid Services (CMS) started endorsing 30-day readmission rates for pneumonia and other medical conditions as quality of care measures and began to impose penalties for hospitals with higher readmission rates [9]. Therefore, reducing readmissions became a major quality improvement focus for the hospitals. Readmission risk predictive modeling can help to identify high-risk patients and thus can assist in prevention strategies to target high-risk patients.

In a systematic review, Weinreich et al. identified 11 models for predicting readmission in patients with pneumonia [10]. Although these models aimed to facilitate risk stratification in pneumonia patients with discriminative capabilities from 0.59 to 0.77, most models were built using traditional statistical approaches, such as regression methods. With the potential for analyzing complex and noisy data, ML can be applied to predict readmission risk. However, the use of machine learning to predict pneumonia-specific readmissions has been limited; current machine learning readmission prediction models for pneumonia, including neural networks and support vector machines, suffered from the issue of poor interpretability or demonstrated low predictive performance by using a simple decision tree [11–14]. As a result, these models have limited utility for informing intervention and high-stake decision-making because policymakers may have challenges in explaining these blackbox models.

Rule ensemble-based learning is a classification algorithm that relies both on a tree-based framework for rule generation and LASSO for rule-pruning and obtaining a compact list of final rules within large datasets for prediction tasks [15–17]. Several works have applied rule-based models to diverse medical datasets for clinical problems, including predicting the onset of diabetes [18], monitoring the prognosis of depression [19], classification of abnormal erythrocytes [20], cancer-type genomic data [21] or multiple sclerosis risk gene [22], and clustering of diabetes population based on a suite of risk factor [23]. The advantage of the rule-based model is its simplicity, with each rule represented in an if–then-else statement involving one or more variables. Thus, the rule learning approach shows the benefit of enhanced interpretability and can be a natural way to alleviate the typical problems of current blackbox models.

The application of rule-based learning in predicting hospital readmission is limited. Only one study applied a rule-based classifier for predicting intensive care unit readmission [24], and none applied the rule-based method in pneumonia. Therefore, to address these gaps, this study focused on a rule-based ensemble model to generate a set of rules for readmission prediction. This study also developed four other ML models, including tree-based models and the LASSO model, and further compared the model performance of the rule-based model with other ML models.

## Methods

### Design and database

This population-based study was conducted using the 2016 Nationwide Readmissions Database (NRD), the part of databases developed for the Agency for Healthcare Research and Quality (AHRQ) Health Care Utilization Project (HCUP). The NRD comprises nationally representative information on hospital readmissions for patients of all ages and all payers. Drawn from the HCUP State Inpatient Databases (SID), the NRD contains inpatient discharges from a variety of hospitals (e.g., community, general acute care, and specialty hospitals). Specifically, the NRD 2016 constructed discharge data sourced from 27 geographically dispersed states, accounting for 57.8% of the total U.S. resident population and 56.6% of all U.S. hospitalizations. A unique, verified patient linkage number is used to track patients across hospitals and is assigned to each admission entry to assist in readmission analysis. The NRD 2016 includes two discharge-level files (Core file and Severity file) and one hospital-level file (Hospital file). The Core file was integrated with the Severity file using a patient linkage number (NRD_VISITLINK) and a unique record identifier (KEY_NRD), and such combined file was then merged with the Hospital file into a unified dataset using the hospital identifier (HOSP_NRD). A total of 17,197,683 discharges were reported in NRD 2016. The study was approved under the exempt category by the University of Houston Institutional Review Board.

### Study population

The study cohort was defined using both demographics and clinical-related criteria from prior work involving readmission analysis in a pneumonia setting [6, 25, 26]. The study cohort included all adult patients (aged ≥ 18 years) who had an index (first) hospitalization from January 1, 2016, through November 31, 2016, with a principal inpatient diagnosis of pneumonia using the International Classification of Diseases, Tenth Revision, codes (J10.0, J10.1, J10.8, J11.0, J11.1, J11.8, J12.0, J12.1, J12.2, J12.3, J12.8, J12.9, J13, J14, J15.x, J16.0, J16.8, J17.0, J17.1, J17.2, J17.3, J17.8, J18.0, J18.1, J18.2, J18.8, J18.9, J69.0, B01.2, B20.6, B25.0, B59) [27, 28] between January 1, 2016, and November 30, 2016. The following hospitalizations were excluded: (1) patients who died during the hospitalization, (2) patients whose index hospitalization was in December because 30-day of follow-up time for readmission analysis is not available, (3) patients with missing data for the length of stay (LOS) or patient linkage number. The flow diagram for the cohort derivation was shown in Additional file 2. A total of 372,293 patient records remained in the dataset.

### Readmission outcomes

The primary outcome was the 30-day all-cause readmissions. It is defined as any hospitalization from any causes within 30 days following discharge of an index admission for pneumonia, which is consistent with previous research [6, 29]. Its goal is intended to broadly consider all subsequent hospitalization within the 30-day period following the index event, regardless of the cause. Readmission was defined as any new admission excluding the index hospitalization regardless of causes within 30 days following an index event. An individual may contribute to multiple index admission events. The first hospitalization for pneumonia was considered an index hospitalization, and any readmission occurring within 30-day was considered a readmission outcome for each index event. For those patients with multiple hospitalizations within 30-day after the index hospitalization, their first hospitalization after the index hospitalization was qualified as their 30-day all-cause readmission outcome. Given the unit of observation was a patient, unique patients identified from these qualified index events with and without readmission were then entered into the analytical cohort.

### Predictor variables

From all available data elements recorded in the NRD data and based on prior NRD studies involving readmission analysis [6, 12], a total of 61 clinically pertinent variables were included for ML model development, including (1) demographics (age, and sex); (2) socioeconomic status (race, expected primary payer, and median household income); (3) healthcare use indicator (number of diagnoses recorded, number of procedures recorded, number of external causes recorded, indicator of emergency service, and indicator of operating room use); (4) comorbid conditions included conditions in the Elixhauser Comorbidity Index [30], and other pneumonia-related conditions operationalized based on literatures [6, 26, 31]; (5) composite score (NRD’s severity measures classified by the 3M All-Patient Refined Diagnosis-Related Group (DRG) severity score), and Elixhauser comorbidity index score [30]); (6) admission/discharge specific factors (discharge month, resident status, and discharge disposition); (7) hospital-level characteristics (control/ownership of hospital, size of hospital, teaching status of hospital, and hospital urban/rural location). Specifically, baseline comorbid conditions were identified based on AHRQ HCUP Elixhauser Comorbidity Software using ICD-10-CM diagnosis codes [32]. Additionally, pneumonia-related conditions were operationalized based on prior work [6, 26, 31] and were identified based on AHRQ HCUP All Patient Refined Diagnosis Related Groups (APR-DRG) Classification Software using medical or surgical APRD_DRG coding system [33]. Procedural classification (minor diagnostic, minor therapeutic, major diagnostic, and major therapeutic) was extracted using the AHRQ HCUP Clinical Classification for Service and Procedures (CCS-Services and Procedures) Software tool based on International Classification of Diseases, Tenth Revision, Procedure Coding System (ICD-10-PCS) [34]. The list of variables and their operational definition are included in the Additional file 2.

### Machine learning model approaches

The study sample was randomly divided into training (50% of the sample) and testing (50% of the sample) sets. The class imbalance was observed in the original data, where the non-readmission class vastly outnumbers the readmission class, resulting in a bias towards the non-readmission class for machine learning classifiers. To improve the machine learning performance, the data resampling method was applied to create more balanced data to better handle the readmission and non-readmission classes [35]. Of all resampling methods, the under-sampling technique is less likely to cause data bias under large datasets and allows computational efficiency, thus was selected to address data imbalance in the current study [36, 37]. The under-sampling balanced the data between majority and minority classes by randomly removing the majority instances (non-readmission class). For our analyses, the under-sampling technique was applied to the training datasets to create balanced training sets for developing the machine learning prediction models. To match the size of the minority class (readmission cases), the under-sampling method is performed with sampling rates of the majority class (non-readmission cases) set at 10%. The testing dataset remains imbalanced as the original data, as reflecting the actual practice, and the performance of these prediction models was then evaluated using the testing data. A rule-based model, tree-based models including decision tree, random forests, eXtreme gradient boosting model (XGBoost), and a LASSO model were constructed using the re-sampled balanced training data set. All models were constructed using the R statistical software (version 3.6.1, RStudio) [45].

#### Rule-based ensemble learning

Prediction rule ensembles (PREs) are a non-parametric exploratory regression method and derive a set of rules for the predictive problem through rule ensemble-based predictive learning [15]. Starting with a tree-based framework to create many candidate rules, PRE applies variable selection method, mainly LASSO, to achieve a compact set of rules in the final model [15]. The interpretability of the model is improved in its simple if–then-else rules. The prediction rule ensemble model was fitted using the R package “pre” [16]. To obtain an optimal set of parameters for model fitting, function “caret” was used to create a tuning grid with a focus on parameters including learnrate (known as boosting parameter), maxdepth (maximal number of conditions per rule), penalty.par.val (penalty parameter λ) and ntrees (number of trees fitted for the initial rule ensemble) [16]. The optimal values for the above parameters were used for model training, and more details could be found in Additional file 1. The important rules discovered from the rule-based ensemble model were visualized, and the decision trees generating these rules were plotted [16]. The importance of variables used to construct the rules in a rule-based ensemble model was also summarized [16].

#### Other machine learning models

A tree-based method is a non-parametric approach that uses a recursive binary partition approach to successively segment the feature space into non-overlapping regions and fit a simple model within each split, and the set of rules for partitioning can be graphically summarized in a tree framework [37, 38]. The decision tree model was implemented in the ‘tree’ package [40], and the tree pruning process was conducted using the inbuilt tenfold cross-validation procedure to avoid overfitting. A random forest is an ensemble of multiple decision tree models by bootstrapping the training samples to build each decision tree and select random subsets of features at each candidate split in the learning process to reduce the correlation between the sampled trees [38]. The random forest was implemented using the ‘Random Forest’ package [41]. The number of decision trees estimated in the random forest was set at 500, which is sufficient for out-of-bag error (OOB) to settle down; the number of candidate variables considered at each split was applied with the default value of $\sqrt{\mathrm{p}}$ (p means the number of predictors); the maximal number of leaf nodes for each tree was tuned in a grid ranging between 10 and 200.

The extreme gradient boosting model (XGBoost) is another tree-based ensemble learner sequentially constructing a series of trees based on information from previously grown trees and combining these “weak learners” to produce a strong classifier [38]. Extreme gradient boosting model was implemented using the ‘xgboost’ package [42]. We set the learning rate of the XGBoost model at 0.01 and the maximum depth of the tree at 4, as the above default values of these parameters are noted with robust performance in various scenarios [43]. A maximum of 10,000 iterations was used; the internal tenfold cross-validation was used to automatically find the best number of boosting rounds. Variable importance was also assessed for the XGBoost model and the random forest model, based on the association between each predictor variable and the response variable. Furthermore, logistic regression involving a LASSO penalty employs a regression-based approach incorporating an L1‐type penalty to the regression objective function. This results in the LASSO obtaining a sparse model by shrinking some parameter coefficients toward 0. The LASSO model was implemented in the Package ‘glmnet’ [44]. A grid of values was chosen for the tuning parameter λ, and a tenfold cross-validation method was performed to select the optimal tuning parameter. The LASSO model performs variable selection and yields only a subset of important features with non-zero coefficients. Details of parameter tuning for all baseline models were found in Additional file 1.

### Performance evaluation of machine learning approaches

The area under the receiver operating characteristics curve (AUROC) is one of the most common performance metrics for comparing models [39]. The AUROC was selected to evaluate the model performance of all modeling approaches in the testing data. AUROC plots the relationship of recall/sensitivity (known as the rate of true positives) against the rate of false positives over a range of threshold levels. The AUROC for machine learning classifiers were compared using the 2-sided DeLong test at a significance level of 0.05 [40].

The area under the precision-recall curves (AUPRC) is informative in understanding binary classification results in imbalanced data [41] and was thus also used to assess model performance in the testing data. The AUPRC depicts the trade-off between precision (ratio of predicted true positives) against the rate of true positives throughout different levels of threshold settings. The AUPRC scores for machine learning classifiers were compared based on absolute difference [42–44]. To inspect the overfitting problem, both AUROC and AUPRC scores were also obtained in training datasets. Both AUROC and AUPRC were obtained using the R package “PRROC” [45].

Also, evaluation metrics, including recall, precision, and F1 score [46], common metrics for imbalanced datasets, were reported (see Additional file 1: eTable 2). To present a performance assessment understandable to clinical stakeholders, accuracy was reported (see Additional file 1: eTable 2). These metrics were calculated based on the confusion matrix using the R package “caret.” [47] The baseline characteristics of the training cohort vs. the testing cohort and by readmission status (yes vs. no) were compared using the χ^2^ test (or Fisher’s exact test) for categorical variables and using the t-test (or a Wilcoxon rank-sum test) for continuous variables. All significance levels were 2-sided, with *P* < 0.05 indicating statistical significance. Analyses were conducted using SAS version 9.3 statistical software (SAS Institute Inc).

## Results

### Study cohort

Among 15,850,247 discharges in NRD 2016, there remained 13,513,774 eligible index admissions among adult patients discharged alive from January 2016 to November 2016. The study population included 372,293 unique patients with a pneumonia diagnosis (Additional file 2: eFigure 1). The overall 30-day readmission rate in this study population was 12.97% (48,280 of 372, 293 patients). From the study cohort, 186,147 of these patients were included in the training set, and the remaining 186,146 were used as the testing sets. The mean (SD) age was 69.04 (16.78) years, 89,184 were men (47.91%), and 130,277 were with Medicare (69.99%) in the training data sets; the mean (SD) age was 69.10 (16.70) years, 89,523 were men (48.09%), and 130,525 were with Medicare (70.12%) in the testing data set. The 30-day readmission rate was similar between the training and testing cohorts, with 12.965% and 12.971% of patients having readmission in the training set and the testing set, respectively. Most characteristics were comparable between the training cohort and testing cohort. Comparisons between those readmitted and those not, as well as between training and testing groups, are found in Additional file 2. After applying the under-sampling method, 48,268 patients were included in the resampled training dataset. Their readmission rate was 50% in the resampled training set, with 24,134 patients having a 30-day readmission outcome. The mean (SD) age was 69.33 (16.29) years, 23,654 were men (49.00%), and 34,659 were with Medicare (71.81%) in the resampled training dataset.

### Comparison of rule-based model and other modelling algorithms

The AUROC of rule-based model in the testing data was 0.6591, which was significantly higher than decision trees (AUROC: 0.5783, *p* value < 0.001), random forest (AUROC: 0.6509, *p* value < 0.01) and LASSO (AUROC: 0.6087, *p* value < 0.001), however, it was significantly lower than XGBoost (0.6606, *p* value = 0.015). The AUPRC of the rule-based model was 0.2146 in the testing dataset, which was better than decision tree (AUPRC: 0.156), random forest (AUPRC: 0.2052), and LASSO (AUPRC: 0.2042); however, the net difference of AUPRC between rule-based model and XGBoost (AUPRC: 0.2147) was small. Details of AUROC and AUPRC for training data are summarized in Table 1. The PR curves for all models were illustrated in the Additional file 1: eFigure 1 and eFigure 2.

**Table 1:** Evaluation of AUROC and AUPRC for all machine learning algorithms

Algorithm	AUROC (95% CI)		*P* value^a^	AUPRC (95% CI)	Net difference^b^
*Testing set*					
Rule-based model	**0.6591 (0.6556–0.6627)**	[Reference]		**0.2146**	[Reference]
Decision tree	0.5783 (0.5751–0.5815)	*P* < 0.001		0.156	− 0.0586
Random forest	0.6509 (0.6473–0.6545)	*P* < 0.01		0.2052	− 0.0094
XGBoost	0.6606 (0.657–0.6641)	0.015		**0.2147**	0.0001
LASSO	0.6087 (0.6053–0.612)	*P* < 0.001		0.2042	− 0.0104
*Training set*					
Rule-based model	0.669 (0.6654–0.6725)	[Reference]		0.219	[Reference]
Decision tree	0.5773 (0.5741–0.5805)	*P* < 0.001		0.1556	− 0.0634
Random forest	0.6558 (0.6522–0.6594)	*P* < 0.001		0.2109	− 0.0081
XGBoost	0.6725 (0.669–0.6761)	*P* < 0.001		0.2279	0.0089
LASSO	0.6062 (0.6029–0.6095)	*P* < 0.001		0.2007	− 0.0183

The best performance model is in bold

ML, machine learning; XGBoost: Extreme Gradient Boosting; AUROC, area under receiver operating curve; LASSO, least absolute shrinkage, and selection operator; AUPRC: area under the precision-recall curve a *p* value is based on the DeLong test for comparison of area under the receiver operating characteristic curves for different models with reference to the rule-based model. b Calculated based on the net difference between all baseline models with reference to the rule-based model

The confusion matrix describing the performance of these models is reported in Additional file 1: eTable 2a. A list of performance metrics, including accuracy, sensitivity (recall), specificity, and precision, were derived from the confusion matrix. They are summarized in Additional file 1: eTables 2b. Further, the F1 score, as a harmonic mean of precision and recall, is a suitable measure for evaluating model performance in imbalanced data and is also summarized in Additional file 1: eTable 2b. Model performance evaluated by other performance metrics (accuracy, sensitivity, specificity, precision, F1 score) varied across models. From the testing data, the rule-based model obtained the highest F1 score, XGBoost showed the highest precision, and accuracy was generally similar across models, with LASSO obtaining the highest. From the training data, XGBoost had the highest precision and F1 score and highest accuracy.

### Most influential predictors

#### Rule-based model

**Fig. 1:**
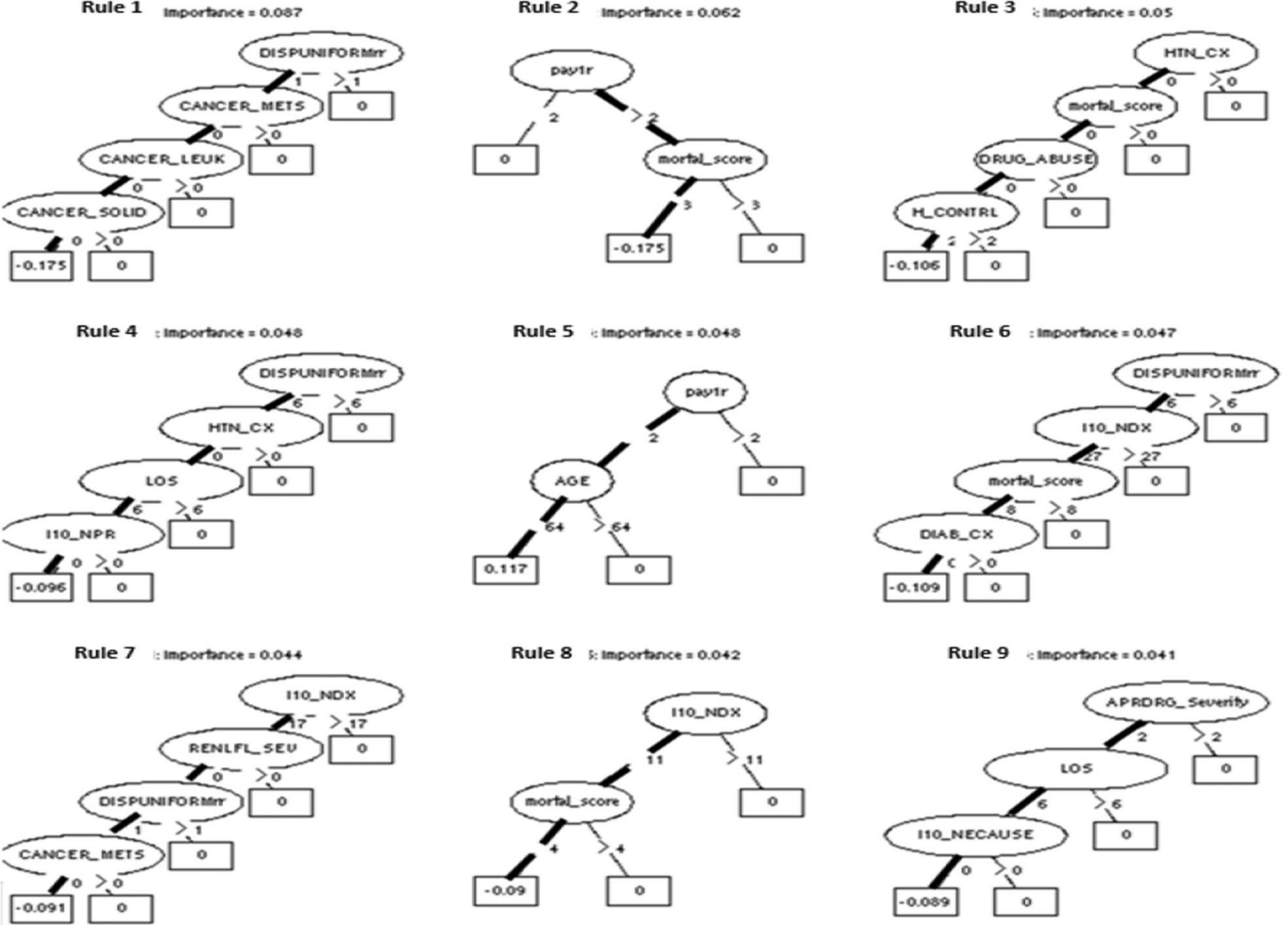
Top nine rules for the rule-based model. *Notes* These plots depict the rules in the final ensemble as a binary tree structure. We chose to present the nine most important rules in the fitted ensemble. This could be obtained using the inbuilt plot method in the PRE algorithm. (1) The risk factors involved in each rule and their cut-off values are automatically determined by the rule-based model. The involved variables and cut-point employed by each rule are highlighted in bolden black: these individuals endorsing these criteria will follow the path of the prediction rule while those individuals not meeting the criteria instead are assigned as zero in the square box. (2) For example, 1st important rule indicates that those satisfying (1) disposition location ≤ 6, (2) metastatic cancer = 0, (3) leukemia = 0, (4) solid tumor = 0, are less likely to have 30-day readmission outcome. Abbreviations and descriptions of data element name: (1) DISPUNIFORMrr: Disposition of patient; (2) CANCER_METS: Elixhauser comorbdity measure of metastatic cancer; (3) CANCER_LEUK: Elixhauser comorbdity measure of leukemia; (4) CANCER_SOLID: Elixhauser comorbdity measure of solid tumor without metastasis, malignant; (5) pay1r: Expected primary payer; (6) mortal_score: Elixhauser Comorbidity Index Score; (7) HTN_CX: Elixhauser comorbdity measure of complicated hypertension; (8) DRUG_ABUSE: Elixhauser comorbdity measure of drug abuse; (9) H_Control: ownership/control of hospital; (10) LOS: length of stay; (11) I10_NPR: number of ICD-10-PCS procedures on this discharge; (12) Age: age at admission; (13) I10_NDX: number of ICD-10-CM diagnoses; (14) DIAB_CX: diabetes, complicated; (15) RENLFL_SEV: Elixhauser comorbdity measure of renal failure, severe; (16) APRDRG_Severity: severity of illness subclass by APR_DRG; (17) I10_NECAUSE: Number of ICD-10-CM External Cause of Morbidity codes on this record

The top rules for the model are presented in Fig. 1. The variables and their cut-off involved in each rule are highlighted in bold black. For ease of illustration, these top rules were also summarized in Table 2. Together, these rules indicate their association with the hospital readmission outcome. For example, rule one finds that if a patient with the disposition of routine type (DISPUNIFORMrr ≤ 1) and had no metastatic cancer (CANCER_METS ≤ 0), leukemia (CANCER_LEUK ≤ 0), or solid cancer (CANCER_SOLID ≤ 0), then the individual is less likely to be readmitted within 30 days after discharge. From the top rules, patient’s comorbidities, illness severity, disposition location, payer type, age, and length of stay were the distinctive variables in differentiating readmission risks. Specifically, it contains rich information for describing a patient’s comorbidities (Rule 1, 3, 4, and 7), the severity of illness (Rule 2, 3, 6, 8), disposition location (Rule 1, 4, 6, and 7) and payer type (Rule 2 and 5).

**Table 2:** The top 9 rules by the rule-based model

Rule 1 (risk decreasing rule)	Rule 6 (risk decreasing rule)
Dispuniformrr ≤ 1 & CANCER_METS ≤ 0 & CANCER_LEUK ≤ 0 & CANCER_SOLID ≤ 0	DISPUNIFORMrr ≤ 6 & I10_NDX ≤ 7 & mortal_score ≤ 8 &DIAB_CX ≤ 0
Rule 2 (risk decreasing rule)	Rule 7 (risk decreasing rule)
pay1r > 2 & mortal_score ≤ 3	I10_NDX ≤ 17& RENLFL_SEV ≤ 0 & DISPUNIFORMrr ≤ 1 & CANCER_METS ≤ 0
Rule 3 (risk decreasing rule)	Rule 8 (risk decreasing rule)
HTN_CX ≤ 0 & mortal_score ≤ 0 & DRUG_ABUSE ≤ 0& H_CONTROL ≤ 2	I10_NDX ≤ 11 & mortal_score ≤ 4
Rule 4 (risk decreasing rule)	Rule 9 (risk decreasing rule)
DISPUNIFORMrr ≤ 6 & HTN_CX ≤ 0 & LOS ≤ 6 & I10_NPR ≤ 0	APRDRG_Severity ≤ 2 & LOS ≤ 6 & I10_NECAUSE ≤ 0
Rule 5 (risk increasing rule)	
pay1r ≤ 2 & age ≤ 64	

(a) 9 top rules identified by the rule-based model, (b) the cut-off values of the variables in the rules were automatically determined by PRE for maximum statistical prediction power

(1) DISPUNIFORMrr: Disposition of patient; (2) CANCER_METS: Elixhauser comorbdity measure of metastatic cancer; (3) CANCER_LEUK: Elixhauser comorbdity measure of leukemia; (4) CANCER_SOLID: Elixhauser comorbdity measure of solid tumor without metastasis, malignant; (5) pay1r: Expected primary payer; (6) mortal_score: Elixhauser Comorbidity Index Score; (7) HTN_CX: Elixhauser comorbdity measure of complicated hypertension; (8) DRUG_ABUSE: Elixhauser comorbdity measure of drug abuse; (9) H_Control: ownership/control of hospital; (10) LOS: length of stay; (11) I10_NPR: number of ICD-10-PCS procedures on this discharge; (12) Age: age at admission; (13) I10_NDX: number of ICD-10-CM diagnoses; (14) DIAB_CX: diabetes, complicated; (15) RENLFL_SEV: Elixhauser comorbdity measure of renal failure, severe; (16) APRDRG_Severity: severity of illness subclass by APR_DRG; (17) I10_NECAUSE: Number of ICD-10-CM External Cause of Morbidity codes on this record

**Fig. 2:**
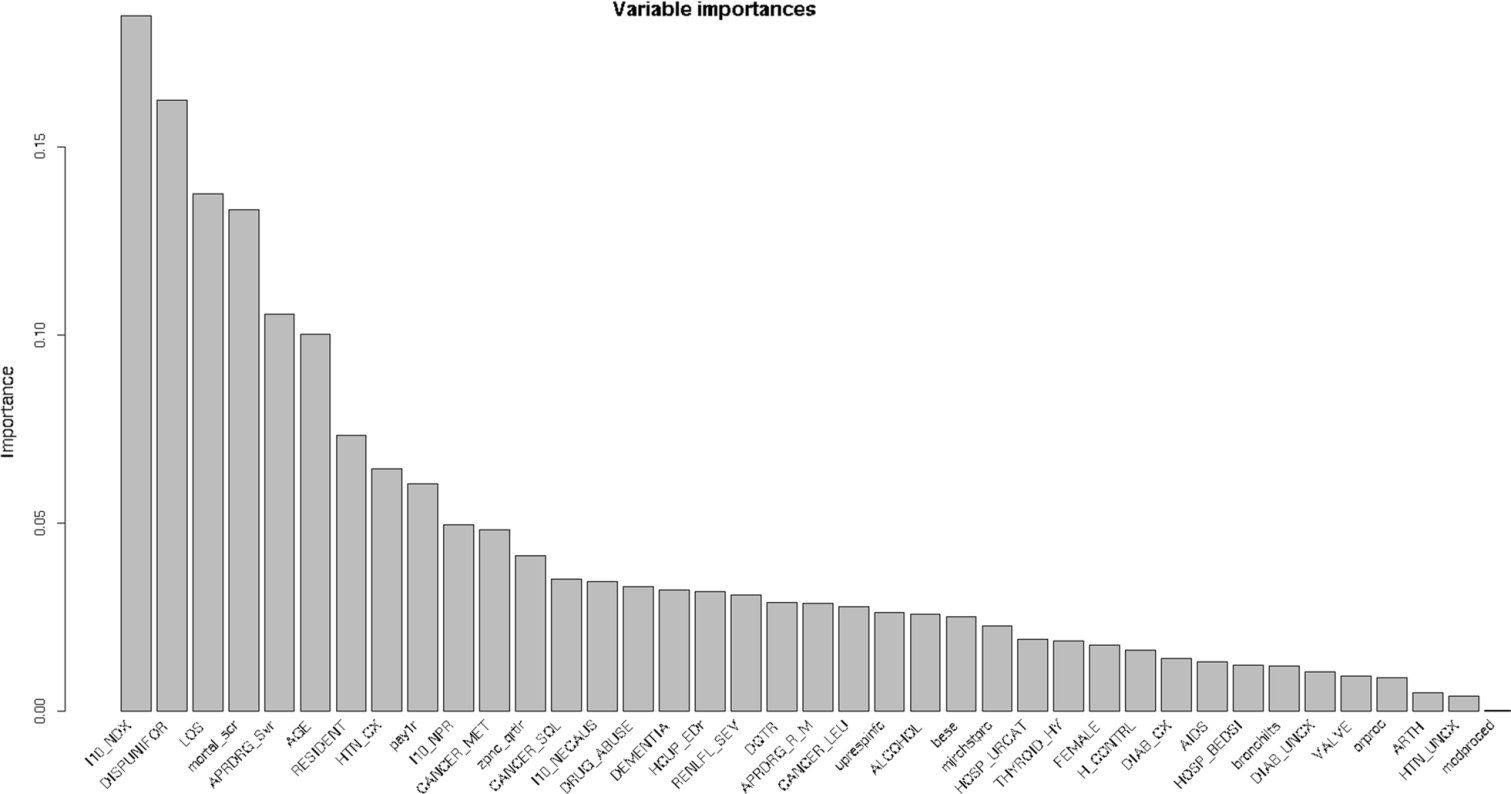
The individual variable importance for the rule-based model. *Notes* The individual variable contributing to the outcome is defined as the sum of the importance of the linear term and the importance of every rule in which the variable appears divided by the total number of conditions in the rule. The 12 most important input variables for predicting the readmission outcome are (1) I10_NDX: Number of ICD-10-CM diagnoses coded on the record; (1) APRDRG_Severity: Severity of Illness Subclass based on 3M All Patient Refined DRG; (2) DISPUNIFORMrr: Disposition of patient; (3) LOS: Length of Stay; (4) mortal_score: Elixhauser Comorbidity Index Score; (5) APRDRG_Severity: All Patient Refined DRG: Severity of Illness Subclass; (6) AGE: the age at admission; (7) Resident: patient as a resident of the State in which he or she received hospital care; (8) HTN_CX: Elixhauser comorbidity measure of Hypertension, complicated; (9) pay1r: Expected primary payer; (10) I10_NPR: number of ICD-10-PCS procedures on this discharge; (11) CANCER_METS: Elixhauser comorbidity measure of Metastatic cancer; and (12) ZIPINC_QRTL: Median household income for patient's ZIP Code (based on current year)

**Fig. 3:**
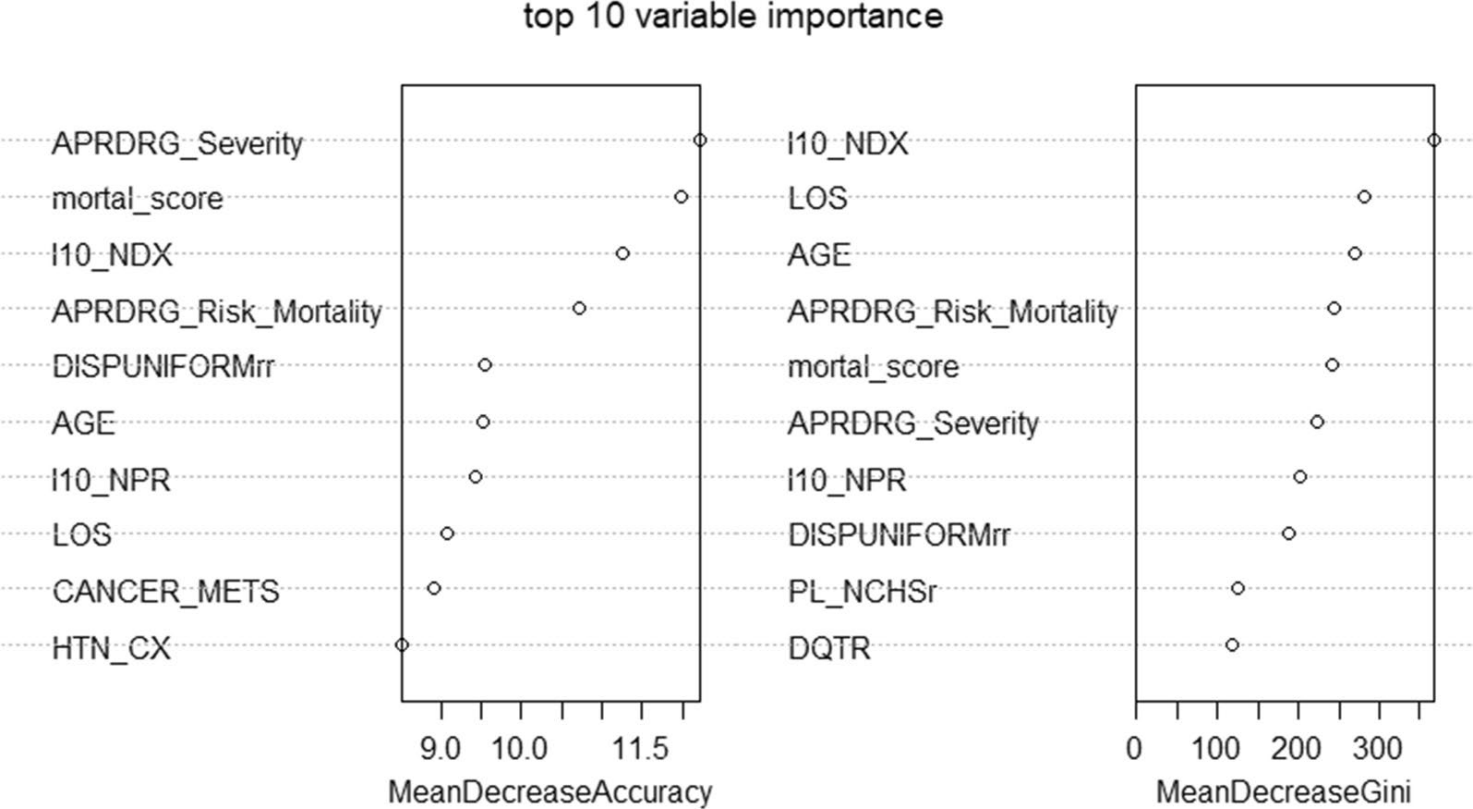
The top 10 most important predictive factors for the random forest. *Notes* A higher "Mean Decrease in Gini" in the x-axis indicates a higher purity (less noise, less bias) contributed by the variable, and higher variable importance). ^1^Variable importance is reported as two measures: it is based on the mean decrease of accuracy in prediction on the out of bag sampled when a given variable is excluded; it is also computed using the mean decrease in Gini index. The variable importance for each variable is expressed relative to the largest. Abbreviations and descriptions of data element name: (1) APRDRG_Severity: Severity of Illness Subclass based on 3M All Patient Refined DRG; (2) mortal_score: Elixhauser Comorbidity Index Score for in-hospital mortality; (3) I10-NDX: Number of ICD-10-CM diagnoses coded on the record; (4) APRDRG_Risk_Mortality: Risk of Mortality Subclass based on 3M All Patient Refined DRG; (5) DISPUNIFORMrr: Disposition of patient; (6) AGE: the age at admission; (7) I10_NPR: Number of procedures coded; (8) LOS: Length of Stay (9) CANCER_METS: Elixhauser comorbidity measure of metastatic cancer; (10) HTN_CX: hypertension, complicated; (11) PL_NCHS: Patient location based on National Center for Health Statistics (NCHS) urban–rural classification scheme for U.S. counties; (12) DQTR: The quarter of Discharge time

**Fig. 4:**
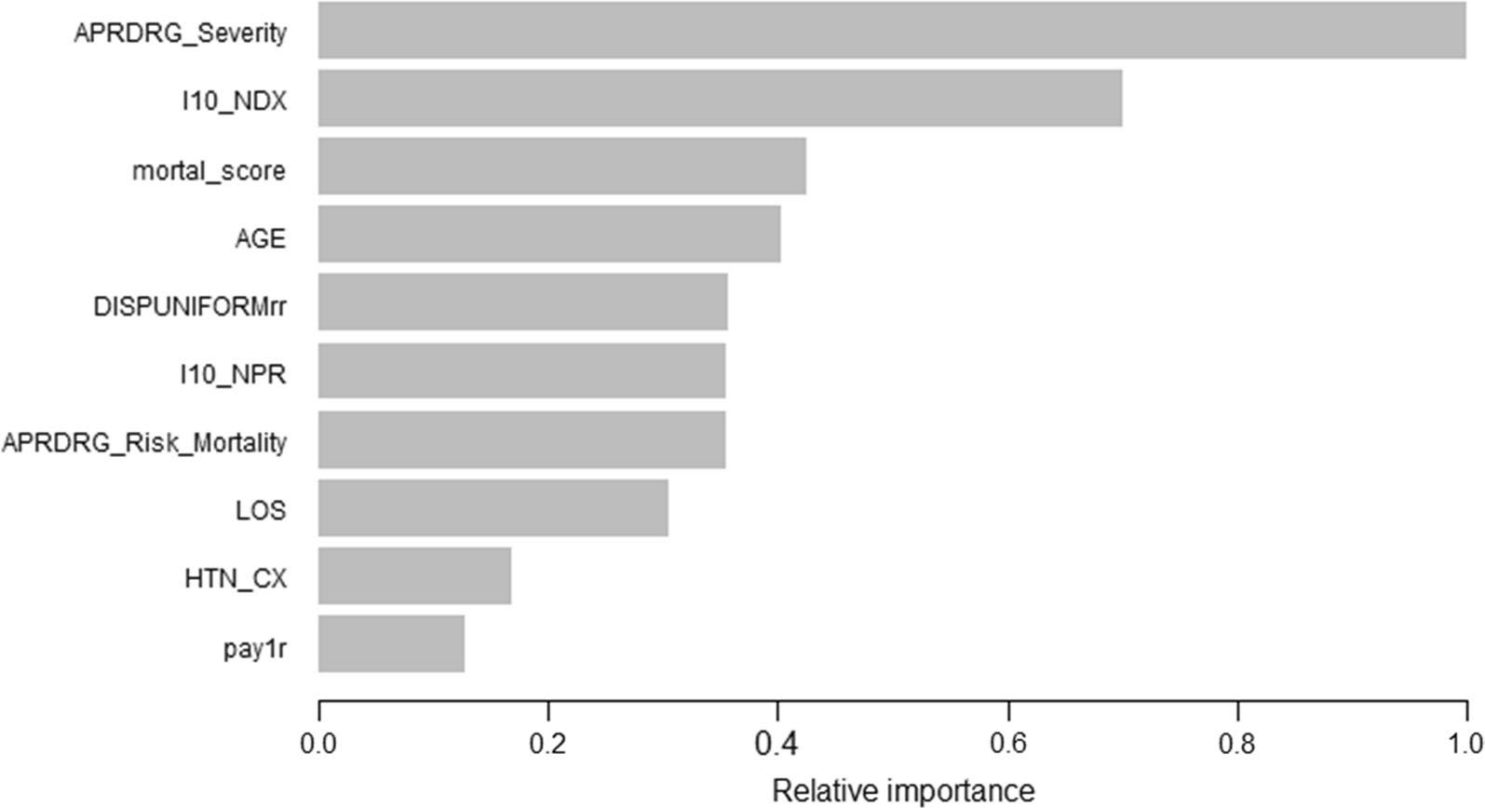
The most important predictor variables in the XGBoost model^1^. *Notes* ^1^The bar graph represents the 10 most important predictor variables in the gradient boosting model sorted by importance. Features are shown ranked in decreasing importance order. The numbers represent the relative importance of each variable. The feature importance is calculated by the feature’s importance contribution relative to the most important feature. The variable importance measure uses the mean decrease in the Gini index to determine the contribution of each predictor variable. Abbreviations and descriptions of data element name: (1) APRDRG_Severity: Severity of Illness Subclass based on 3M All Patient Refined DRG; (2) I10-NDX: Number of ICD-10-CM diagnoses coded on the record; (3) mortal_score: Elixhauser Comorbidity Index Score; (4) AGE: the age at admission; (5) DISPUNIFORMrr: Disposition of patient; (6) I10_NPR: Number of procedures coded; (7) APRDRG_Risk_Mortality: Risk of Mortality Subclass based on 3M All Patient Refined DRG; (8) LOS: Length of Stay; (9) HTN_CX: Elixhauser comorbidity measure of complicated hypertension; (10) pay1r: Expected primary payer

The relative importance of each variable is shown in Fig. 2. The analysis of variable importance had considerable overlap in the variables involved in the top rules. Consistently, these leading risk factors from the top 9 identified rules were also found to have the most predictive power for readmission risk based on their variable importance. The 12 most important input variables for predicting the readmission outcome based on rule-based learning are (1) number of ICD-10-CM diagnoses, (2) disposition of patient, (3) length of stay, (4) Elixhauser index score, (5) severity of illness, (6) age, (7) resident status, (8) comorbidity measure of complicated hypertension, (9) payer type, (10) number of procedures, (11) metastatic cancer, and (12) median household income for patient’s zip code. These leading risk factors detected from the identified rules were also consistent with most of the significant risk factors identified from the random forest and XGBoost model, and LASSO (Figs. 3, 4, Table 3).

**Table 3:** Features with variable importance in descending order of non-zero coefficient effect size selected by LASSO logit regression

Variables	Coefficients
APRDRG_Severity	0.1848
RENLFL_SEV	0.1683
CANCER_METS	0.1363
HTN_CX	0.1025
I10_NDX	0.0261
I10_NPR	0.0048
mortal_score	0.0048
LOS	0.0099
DISPUNIFORMrr	0.0014
APRDRG_Risk_Mortality	0.0009

LASSO model shrinks some coefficient estimates towards zero. Here, we see that only 9 of the 61 coefficient estimates are selected by LASSO Model. Variable importance is ranked by the absolute value of the coefficient for the LASSO model

(1) APRDRG_Severity: Severity of Illness Subclass based on 3M All Patient Refined DRG; (2) RENLFL_SEV: Elixhauser comorbidity measure of moderate and severe renal failure; (3) CANCER_METS: Elixhauser comorbidity measure of metastatic cancer; (4) HTN_CX: Elixhauser comorbidity measure of Hypertension, complicated; (5) I10_NDX: Number of ICD-10-CM diagnoses coded on the record; (6) I10_NPR: Number of procedures coded; (7) mortal_score: Elixhauser Comorbidity Index Score for in-hospital mortality; (8) LOS: Length of Stay; (9) DISPUNIFORMrr: Disposition of patient; (10) APRDRG_Risk_Mortality: Risk of Mortality Subclass based on 3M All Patient Refined DRG

#### Random forest

The variable importance for readmission using random forest is shown in Fig. 1. The top 10 variables with the largest mean decrease in accuracy are (1) Severity of Illness (APRDRG_Severity); (2) Elixhauser index score (mortal_score); (3) Number of ICD-10-CM diagnoses coded on the record (I10_NDX); (4) Risk of Mortality (APRDRG_Risk_Mortality); (5) Disposition of patient (DISPUNIFORMrr); (6) Age at admission (AGE); (7) Number of procedures coded (I10_NPR); (8) Length of Stay (LOS); (9) Elixhauser comorbidity measure of metastatic cancer (CANCER_METS); (10) Elixhauser comorbidity measure of hypertension, complicated (HTN_CX). The ten variables with the largest mean decrease in Gini index are (1) the Number of ICD-10-CM diagnoses coded on the record (I10_NDX); (2) Length of Stay (LOS); (3) the age at admission (AGE); (4) Risk of Mortality (APRDRG_Risk_Mortality); (5) Elixhauser index score (mortal_score); (6) Severity of Illness (APRDRG_Severity); (7) Number of procedures coded (I10_NPR); (8) Disposition of patient (DISPUNIFORMrr); (9) Patient location (PL_NCHS); (10) The quarter of discharge time (DQTR). The results indicate that across two measures of the ten most important predictive variables, the most important predictive variables are (1) Severity of Illness (APRDRG_Severity); (2) Elixhauser index score (mortal_score); (3) Number of ICD-10-CM diagnoses coded on the record (I10_NDX); (4) Risk of mortality (APRDRG_Risk_Mortality), (5) Disposition of patient (DISPUNIFORMrr); (6) Age, (7) Number of procedures coded (I10_NPR); and (8) Length of stay (LOS).

#### XGBoost model

The variable importance based on the XGBoost model is shown in Fig. 4. The variable importance measure uses the mean decrease in the Gini index to determine the contribution of each predictor variable. The ten most important predictor variables in the XGBoost model were (1) Severity of Illness Subclass based on 3M All Patient Refined DRG (APRDRG_Severity); (2) Number of ICD-10-CM diagnoses coded on the record (I10-NDX); (3) Elixhauser index score (mortal_score); (4) the age at admission (AGE); (5) Disposition of patient (DISPUNIFORMrr); (6) Number of procedures coded (I10_NPR); (7) Risk of Mortality Subclass based on 3M All Patient Refined DRG (APRDRG_Risk_Mortality); (8) Length of Stay (LOS); (9) Elixhauser comorbidity measure of complicated hypertension (HTN_CX); and (10) Expected primary payer (pay1r).

#### LASSO model

The variables selected by the LASSO model in terms of their coefficient estimates are shown in Table 3. The LASSO yields a sparse model as it shrinks the coefficient estimates of other variables towards zero, thus involving only a subset of the variables. The LASSO model with λ chosen by cross-validation contains only nine variables. These variables in terms of variable importance for readmission prediction generated by the LASSO algorithm were (1) Severity of Illness based on APR_DRG (APRDRG_Severity), (2) Elixhauser Comorbidity measure of moderate and severe renal failure (RENLFL_SEV), (3) Elixhauser comorbidity measure of metastatic cancer (CANCER_METS), (4) Elixhauser comorbidity measure of hypertension, complicated (HTN_CX). (5) Risk of Mortality based on APR_DRG (APRDRG_Risk_Mortality), (6) Number of ICD-10-CM diagnoses coded on the record (I10_NDX), (7) Length of stay (LOS), (8) Number of procedures coded (I10_NPR), (9) Elixhauser index score (mortal_score), and (10) Disposition of patient (DISPUNIFORMrr).

## Discussion

This study successfully utilized the rule-based ensemble learning algorithm and other ML algorithms to predict readmission in a pneumonia setting. The analyses based on the US nationally representative data of hospitalization records found that the rule-based learning method has comparable performance to tree-based ensemble methods for readmission prediction in a pneumonia setting. However, XGBoost outperforms the rule-based methods for pneumonia-specific readmission. Comorbidity, illness severity, disposition location, payer type, age, and length of stay were prominent variables identified from the top rules. These high predictive variables are corroborated by comparisons across multiple machine learning methods developed in this study.

Our study adds to a body of literature showing the application of machine learning approaches for the task of hospital readmission risk prediction. In a systematic review of 43 studies, Huang et al. found tree-based methods and neural networks to the common machine learning for hospital readmission prediction [48]. This proposed study effectively developed a rule-based ensemble model to predict the risk for 30-day all-cause readmission, enriching the existing ML toolbox for the clinical risk prediction problems of hospital readmission. Specifically, in a pneumonia setting, previous neural network or support vector machine models are black-box models, limiting their use in the clinical setting [11, 12, 14]. Our rule-based model complements the above state-of-art machine learning models with improved interpretability and shows that these generated if–then-else rules are with potential to assist clinical decision-making.

In the pioneering work, Fokkema showed that a rule-based model could achieve comparable performance with random forest and LASSO by applying survey data to predict depression [17]. Judged by most evaluation metrics, the rule-based model outperformed decision tree, LASSO, and random forest, whereas it did not fare better than the XGBoost model. This is consistent with the algorithm of XGBoost models [49] as it sequentially fits a tree with errors corrected from already grown trees and implements a one-hot encoding-based tree splits approach and thereby achieving high predictive accuracy with carefully tuned parameters. Future studies may apply natural language processing techniques to extract unstructured features to refine these models and improve prediction. In this study, the AUROC and AUPRC were used as the main metrics for model evaluation. The precision-recall curve (AUPRC) is considered as a robust measure in unbalanced data because it focuses on identifying the correct prediction of minority class and is more sensitive to false positives, a more meaningful concept in imbalanced data [41, 50, 51]. While the ROC curve is popular in evaluating the binary classifiers, it leads to the wrong interpretation of specificity in the context of imbalanced data [41, 50, 51]. Also, the accuracy can be misleading under imbalanced data (e.g., when the outcome is rare, blindly predicting all results as majority class can result in high accuracy) [52]. It is worth mentioning that the down-sampling technique applied was able to balance the data and effectively improve the classifiers’ predictive performance. Furthermore, despite interest in the comparative performance of ML vs. traditional regression methods for readmission [53, 54] and other outcomes [55], a comparison of the performance between different machine learning methods still unexplored for specific clinical problems. Future work involving the comparison of rule-based models and other machine learning methods could guide choosing an optimal algorithm in real-life situations.

From the rule-based model, top rules adequately captured patients’ comorbidities, composite severity score, disposition location, age, and length of stay. This matches well with the results in the literature showing comorbidities and illness severity [56], discharge location [57], age [31], and length of stay [58–60] contribute to readmission risk in pneumonia patients. These risk factors are also corroborated by tree-based and LASSO models as variables with high importance, providing validation to the rule-based model. Interestingly, social factors, such as neighborhood income and residence type, emerged with high variable importance in the rule-based learning despite the fact that they are not present in the top derived rules. Downing and colleagues showed that these variables, which manifest an individual’s access to care, social support, and socioeconomic disadvantage, potentially contribute to readmission risk [61]. Inclusion of more rules would conceivably capture more highly influential variables to predict readmission risk. Furthermore, both rule-based analysis and XGBoost detected unreported social factors, namely, payer type contributing to pneumonia-specific readmission risk, and this remains to be investigated.

The interpretability of the rule-based model has been demonstrated by prior work in predicting substance use [17], predicting bipolar disorder [62], or assessing risk for eating disorders [63]. The benefits in terms of model interpretability are highlighted here by the compact if–then-else rules generated by the rule-based model in explaining readmission risk. For this, the rule-based model is particularly attractive for the clinical purpose of readmission risk stratification. First, the rule-based model takes a compact list of variables to define the top rules through regularization methods [15, 16], enabling clinicians can use fewer variables to distinguish readmission risk. Second, rule-based methods are capable of capturing interaction effects amongst variables through automatic search approaches [15, 16] and therefore can be considered as an exploratory tool for identifying undetected interaction effects. Considering the clinical purpose for identifying high-risk populations to facilitate early intervention, the rule-based model enables the possibility of explaining the readmission risk prediction in terms of top rules and could empower clinicians to screen high-risk patients based on their data for efficient use of resources.

### Strengths and limitations

The strength of the study includes the novelty of using rule ensemble approaches for predicting readmission, advancing the existing ML toolbox for the readmission risk prediction problem. The rule-based model constructed in the current study holds a promise to become a template for others to develop an interpretable model for readmission problems and other clinical problems. Second, the study used a comprehensive set of evaluation metrics, and specifically, this study incorporates the AUPRC, and F1 scores, shedding insights on the model performance in an imbalanced dataset. Thirdly, the high generalizability of predictors derived for pneumonia-specific readmission as the data source is nationally representative of various geographic regions of the US.

This study has some limitations. First, the NRD data source lacks clinical factors such as health status [64, 65] and socioeconomic factors [66]. Second, the obtained AUPRC is low and typically lacks high precision. Precision represents the proportion of times correctly classifying a patient as readmitted when he/she was readmitted in actuality. Based on others’ related work, lessening the threshold of classifying readmission may address the problem of low precision [64]. However, the precision obtained here is comparable to previous work on the ML-based readmission model [67]. This together indicates the complex nature underlying the readmission problem, supporting the efforts for querying unstructured data for clinically granular data as well as incorporating social determinant-related data to increase the predictive performance of the readmission problem [65, 66]. Thirdly, while the study demonstrates that the model prediction performance is maintained in an internal validation dataset, adopting the machine learning approach among an external dataset would likely be required for the implementation of the model. Fourthly, based on previous work from others, using the neural network method or support vector machine readmission is also common in readmission prediction [48]; further performance gains for predicting readmission may be achieved through these methods, and additional work could compare rule ensemble-based methods with these models. Next, our data source contained a patient population with pneumonia diagnosis in 2016, which limits the ability to address emerging infections such as COVID 19. Future work in the prediction of pneumonia-related readmission needs to account for the infection with COVID 19. Finally, while our ML models evaluated readmission risk in patients with pneumonia, future studies will need to develop ML models in the subgroups of patients to obtain more precise information for readmission risk stratification.

## Conclusions

Using a large national readmission database, this study found that the performance of these machine learning methods varied in predicting pneumonia-specific readmission outcomes. Rule-based models outperformed most machine learning models but did not outperform XGBoost. The risk factors involved in the important rules included comorbidities, illness severity, disposition location, payer type, age, and length of stay. Other ML models also validated these above variables as high important predictors. Comparative performance of the rule-based method and other ML methods warrants further evaluation of other health outcomes.

## Supplementary Information

**Additional file 1**. Model Development Parameters and Performance Metrics.

**Additional file 2**. Study Cohort Derivation and Characteristics.

## Data Availability

Data used in the current study from the Nationwide Readmissions Database (NRD), available in https://www.hcup-us.ahrq.gov/nrdoverview.jsp. The data are available from the corresponding author on reasonable request.

## Abbreviations

NN: Neural networks
AUROC: Area under the receiver operating curves
AUPRC: Area under the precision-recall curves
LASSO: Least absolute shrinkage and selection operator
ML: Machine learning
NLP: Natural language processing
SVM: Support vector machine
RF: Random forest
XGBoost: Extreme gradient boosting machine
DT: Decision tree
CMS: Centers for Medicare and Medicaid Services
ML: Machine Learning
ICU: Intensive Care Unit
