# Supplementary material for: Machine learning methods to predict 30-day hospital readmission outcome among US adults with pneumonia: analysis of the national readmission database
Source: BMC Med Inform Decis Mak. 2022 Nov 9;22:288. doi: 10.1186/s12911-022-01995-3 (PMC9643900; doi:10.1186/s12911-022-01995-3)

**ADDITIONAL FILE 1**

**MODEL DEVELOPMENT PARAMETERS AND PERFORMANCE METRICS**

**eTable 1 Parameter tuning process for all models**

**eTable 2a. Confusion Matrix of All Models and Calculation of Evaluation Metric**

**eTable 2b Reporting of evaluation metrics for all models**

**eFigure 1 Precision recall curves for all models in the testing data**

**eFigure 2 Precision recall curves for all models in the training data**

**Additional file 1: eTable 1. Hyper-parameters Tuning Setting for All Models**

| **eTable 1. Hyper-parameters of All Algorithms** | | | |
| --- | --- | --- | --- |
| **Parameter name** | **Parameter description** | **Distribution and search range (Procedures)** | **Best parameter** |
| **Rule-Based Algorithm** | | |  |
| learnrate | learning rate or boosting parameter | A manner of customized grid search with the value of rate ranging from 0.01 to 0.1 (Using automated function “caret”) | 0.04 |
| ntrees | number of trees | A manner of customized grid search with the value of rate ranging from 500 to 1,250 (Using automated function “caret”) | 1,000 |
| maxdepth | maximum number of conditions in rules | A manner of customized grid search with the value of length ranging from 1 to 5 (Using automated function “caret”) | 4 |
| λ (penalty.par.val) | the penalty parameter | A manner of customized grid search (Using automated function “caret”) | lambda.min |
| **XGBoost Algorithm** | | |  |
| nrounds | the maximal number of iterations | 10 fold cross validation within a maximum of 10, 000 iterations | 1,100 |
| eta | step size of each boosting step, controls learning rate | Default parameters value | 0.01 |
| max_depth | maximum depth of the tree | Default parameters value | 4 |
| **Random Forests** | | |  |
| *ntrees* | *number of trees* | *as number of trees grow, the OOB error drops down and become constant unable to improve the error after 500 trees* | *500* |
| *mtry* | *the average number of terminal nodes* | *Default parameters value=√p (square root of the number of p features)* | *7* |
| *maxnodes* | *Maximum number of terminal nodes trees in the forest can have* | *A manner of customized grid search with the value of ranging from 10 to 200* | *100* |
| **LASSO** | | |  |
| λ | the penalty parameter | A manner of grid search with λ ranging from 10^ (-10) to 10^10 (Using 10-fold cross-validation) | 0.0024 |
| **Decision Trees Algorithm** | | |  |
| size | number of terminal nodes of each tree considered | the classification error rate to guide the cross-validation and pruning process | 2 |

**eTable 2a. Confusion Matrix and Calculation of Other Evaluation Metrics**

The measures of model performance included evaluation metrics directly derived from the

confusion matrix, including (1) accuracy, (2) sensitivity, (3) specificity.

Other evaluation metrics, including (4) precision, (5) recall and (6) F1 score were measured

based on the items within confusion matrix.

Evaluation metrics are selected as the popular precision (P), recall (R) and F1-measure (F1) as illustrated by equation below. To make our experiments comparable with baselines, all the results are evaluated using this script.

$$Precision (P)=\frac{true positive (tp)}{true positive \left( tp \right)+false positive (fp)}$$

$$Recall (R)=\frac{true positive (tp)}{true positive \left( tp \right)+false negative (fn)}$$

$$F1 Score (f1)=\frac{2*Precision \left( P \right)*Recall(R)}{Precision \left( P \right)+Recall (R)}$$

1. **Rule-Fit Model**

***Testing set:***

Confusion Matrix and Statistics

Reference

Prediction 0 1

0 95460 8685

1 66512 15490

Accuracy : 0.596

95% CI : (0.5938, 0.5983)

No Information Rate : 0.8701

P-Value [Acc > NIR] : 1

Kappa : 0.1141

Mcnemar's Test P-Value : <2e-16

Sensitivity : 0.64074

Specificity : 0.58936

Pos Pred Value : 0.18890

Neg Pred Value : 0.91661

Prevalence : 0.12987

Detection Rate : 0.08321

Detection Prevalence : 0.44052

Balanced Accuracy : 0.61505

'Positive' Class : 1

**Other Evaluation Metrics**

$Precision (P)=\frac{15490}{15490+66512}$ *≈0.1889*

$Recall \left( R \right)=\frac{15490}{15490+8685}\approx0.$*6407*

$$F1 Score \left( f1 \right)=\frac{2*0.1889*0.6407}{0.1889+0.6407}\approx0.2918$$

***Training set:***

Confusion Matrix and Statistics

Reference

Prediction 0 1

0 95378 8463

1 66663 15642

Accuracy : 0.5964

95% CI : (0.5942, 0.5986)

No Information Rate : 0.8705

P-Value [Acc > NIR] : 1

Kappa : 0.1171

Mcnemar's Test P-Value : <2e-16

Sensitivity : 0.64891

Specificity : 0.58860

Pos Pred Value : 0.19005

Neg Pred Value : 0.91850

Prevalence : 0.12950

Detection Rate : 0.08403

Detection Prevalence : 0.44215

Balanced Accuracy : 0.61876

'Positive' Class : 1

**Other Evaluation Metrics**

$Precision (P)=\frac{15642}{15642+66663}$ *≈0.1900*

$$Recall \left( R \right)=\frac{15642}{15642+8463}\approx0.6489$$

$$F1 Score \left( f1 \right)=\frac{2*0.1900*0.6489}{0.1900+0.6489}\approx0.2940$$

1. **Decision Trees**

***Testing sets:***

Confusion Matrix and Statistics

Reference

Prediction 0 1

0 73847 7344

1 88038 16918

Accuracy : 0.4876

95% CI : (0.4853, 0.4899)

No Information Rate : 0.8697

P-Value [Acc > NIR] : 1

Kappa : 0.0636

Mcnemar's Test P-Value : <2e-16

Sensitivity : 0.69730

Specificity : 0.45617

Pos Pred Value : 0.16119

Neg Pred Value : 0.90955

Prevalence : 0.13034

Detection Rate : 0.09089

Detection Prevalence : 0.56383

Balanced Accuracy : 0.57674

'Positive' Class : 1

**Other Evaluation Metrics**

$$Precision (P)=\frac{16918}{16918+88038}\approx0.1612$$

$$Recall (R)=\frac{16918}{16918+7344}\approx0.6973$$

$$F1 Score \left( f1 \right)=\frac{2*0.1612*0.6973}{0.1612+0.6973}\approx0.2619$$

***Training sets:***

Confusion Matrix and Statistics

Reference

Prediction 0 1

0 73751 7079

1 88377 16939

Accuracy : 0.4872

95% CI : (0.4849, 0.4895)

No Information Rate : 0.871

P-Value [Acc > NIR] : 1

Kappa : 0.0656

Mcnemar's Test P-Value : <2e-16

Sensitivity : 0.7053

Specificity : 0.4549

Pos Pred Value : 0.1608

Neg Pred Value : 0.9124

Prevalence : 0.1290

Detection Rate : 0.0910

Detection Prevalence : 0.5658

Balanced Accuracy : 0.5801

'Positive' Class : 1

**Other Evaluation Metrics**

$$Precision (P)=\frac{16939}{16939+88377}\approx0.1608$$

$$Recall (R)=\frac{16939}{16939+7079}\approx0.7053$$

$$F1 Score \left( f1 \right)=\frac{2*0.1608*0.7053}{0.1608+0.7053}\approx0.2619$$

1. **Random Forest**

***Testing sets:***

Confusion Matrix and Statistics

Reference

Prediction 0 1

0 95787 9367

1 64098 14895

Accuracy : 0.6053

95% CI : (0.6031, 0.6076)

No Information Rate : 0.8697

P-Value [Acc > NIR] : 1

Kappa : 0.1113

Mcnemar's Test P-Value : <2e-16

Sensitivity : 0.61392

Specificity : 0.60405

Pos Pred Value : 0.18856

Neg Pred Value : 0.91258

Prevalence : 0.13034

Detection Rate : 0.08002

Detection Prevalence : 0.42436

Balanced Accuracy : 0.60899

'Positive' Class : 1

**Other Evaluation Metrics**

$Precision (P)=\frac{14895}{14895+64098}$ $\approx0.1886$

$$Recall (R)=\frac{14895}{14895+9367}\approx0.6139$$

$$F1 Score \left( f1 \right)=\frac{2*0.1886*0.6139}{0.1886+0.6139}\approx0.2885$$

***Training sets:***

Confusion Matrix and Statistics

Reference

Prediction 0 1

0 99890 7676

1 62238 16342

Accuracy : 0.6244

95% CI : (0.6222, 0.6266)

No Information Rate : 0.871

P-Value [Acc > NIR] : 1

Kappa : 0.1507

Mcnemar's Test P-Value : <2e-16

Sensitivity : 0.68041

Specificity : 0.61612

Pos Pred Value : 0.20797

Neg Pred Value : 0.92864

Prevalence : 0.12903

Detection Rate : 0.08779

Detection Prevalence : 0.42214

Balanced Accuracy : 0.64826

'Positive' Class : 1

**Other Evaluation Metrics**

$Precision (P)=\frac{16342}{16342+62238}$ $\approx0.2080$

$$Recall (R)=\frac{16342}{16342+7676}\approx0.6804$$

$$F1 Score \left( f1 \right)=\frac{2*0.2080*0.6804}{0.2080+0.6804}\approx0.3186$$

1. **XGboost**

***Testing sets:***

Confusion Matrix and Statistics

Reference

Prediction 0 1

0 96446 8950

1 65439 15312

Accuracy : 0.6004

95% CI : (0.5981, 0.6026)

No Information Rate : 0.8697

P-Value [Acc > NIR] : 1

Kappa : 0.114

Mcnemar's Test P-Value : <2e-16

Sensitivity : 0.63111

Specificity : 0.59577

Pos Pred Value : 0.18962

Neg Pred Value : 0.91508

Prevalence : 0.13034

Detection Rate : 0.08226

Detection Prevalence : 0.43380

Balanced Accuracy : 0.61344

'Positive' Class : 1

**Other Evaluation Metrics**

$Precision (P)=\frac{15312}{15312+65439}$ $\approx0.1896$

$Recall (R)=\frac{15312}{15312+8950}\approx$0.6311

$$F1 Score \left( f1 \right)=\frac{2*0.1896*0.6311}{0.1896+0.6311}\approx0.2916$$

***Training sets:***

Confusion Matrix and Statistics

Reference

Prediction 0 1

0 14832 8247

1 9186 15771

Accuracy : 0.6371

95% CI : (0.6328, 0.6414)

No Information Rate : 0.5

P-Value [Acc > NIR] : <2.2e-16

Kappa : 0.2742

Mcnemar's Test P-Value : 1.21e-12

Sensitivity : 0.6566

Specificity : 0.6175

Pos Pred Value : 0.6319

Neg Pred Value : 0.6427

Prevalence : 0.5000

Detection Rate : 0.3283

Detection Prevalence : 0.5195

Balanced Accuracy : 0.6371

'Positive' Class : 1

**Other Evaluation Metrics**

$Precision (P)=\frac{15771}{15771+9186}$ $\approx0.6319$

$Recall (R)=\frac{15771}{15771+8247}\approx$0.6566

$$F1 Score \left( f1 \right)=\frac{2*0.6319*0.6566}{0.6319+0.6566}\approx0.6440$$

**(e) LASSO**

***Testing sets:***

Confusion Matrix and Statistics

Reference

Prediction 0 1

0 101296 10105

1 60589 14157

Accuracy : 0.6202

95% CI : (0.618, 0.6224)

No Information Rate : 0.8697

P-Value [Acc > NIR] : 1

Kappa : 0.111

Mcnemar's Test P-Value : <2e-16

Sensitivity : 0.58351

Specificity : 0.62573

Pos Pred Value : 0.18940

Neg Pred Value : 0.90929

Prevalence : 0.13034

Detection Rate : 0.07605

Detection Prevalence : 0.40154

Balanced Accuracy : 0.60462

'Positive' Class : 1

**Other Evaluation Metrics**

$Precision (P)=\frac{14157}{14157+60589}$ $\approx0.1894$

$$Recall (R)=\frac{14157}{14157+10105}\approx0.5835$$

$$F1 Score \left( f1 \right)=\frac{2*0.1894*0.5835}{0.1894+0.5835}\approx0.2860$$

***Training sets:***

Confusion Matrix and Statistics

Reference

Prediction 0 1

0 101106 9689

1 61022 14329

Accuracy : 0.6201

95% CI : (0.6179, 0.6223)

No Information Rate : 0.871

P-Value [Acc > NIR] : 1

Kappa : 0.1153

Mcnemar's Test P-Value : <2e-16

Sensitivity : 0.59659

Specificity : 0.62362

Pos Pred Value : 0.19016

Neg Pred Value : 0.91255

Prevalence : 0.12903

Detection Rate : 0.07695

Detection Prevalence : 0.40480

Balanced Accuracy : 0.61011

'Positive' Class : 1

**Other Evaluation Metrics**

$Precision (P)=\frac{14329}{14329+61022}$ $\approx0.1902$

$$Recall (R)=\frac{14329}{14329+9689}\approx0.5966$$

$$F1 Score \left( f1 \right)=\frac{2*0.1902*0.5966}{0.1902+0.5966}\approx0.2884$$

**eTable 2b. Summary of Above Performance Metrics of ML Models**

| **eTable 2b. Performance Metrics of ML Models for Predicting 30-day Readmission in Pneumonia** | | | | | |  |
| --- | --- | --- | --- | --- | --- | --- |
| **Algorithm** | **Accuracy (95% CI)** | **Sensitivity (Recall)** | **Specificity** | **Precision** | **F1 Score** |  |
|  |  |  |  |  |  |  |
| **Testing sets** | | | | | |  |
| Rule-based model^1^ | 0.596 (0.5938-0.5983) | 0.6407 | 0.5894 | 0.1889 | 0.2918 |  |
| Decision Tree^2^ | 0.4876 (0.4853- 0.4899) | 0.6973 | 0.4562 | 0.1612 | 0.2619 |  |
| Random Forest^3^ | 0.6053 (0.6031-0.6076) | 0.6139 | 0.6041 | 0.1886 | 0.2885 |  |
| XGBoost^4^ | 0.6004 (0.5981-0.6026) | 0.6311 | 0.5958 | 0.1896 | 0.2916 |  |
| LASSO^5^ | 0.6202 (0.618-0.6224) | 0.5835 | 0.6257 | 0.1894 | 0.2860 |  |
| **Training sets** |  |  |  |  |  |  |
| Rule-based model^1^ | 0.5964 (0.5942-0.5986) | 0.6489 | 0.5886 | 0.1900 | 0.2940 |  |
| Decision Tree^2^ | 0.4872 (0.4849- 0.4895) | 0.7053 | 0.4549 | 0.1608 | 0.2619 |  |
| Random Forest^3^ | 0.6244 (0.6222-0.6266) | 0.6804 | 0.6161 | 0.2080 | 0.3186 |  |
| XGBoost^4^ | 0.6371 (0.6328-0.6414) | 0.6566 | 0.6175 | 0.6319 | 0.6440 |  |
| LASSO^5^ | 0.6201 (0.6179-0.6223) | 0.5966 | 0.6236 | 0.1902 | 0.2884 |  |
| **Abbreviations**: ML, machine learning; XGBoost, Extreme Gradient Boosting; AUC, area under curve; LASSO, least absolute shrinkage and selection operator; TP, true positive; TN, true negative; FP, false positive; FN, false negative | | | | | |  |
| Notes: a. Accuracy=(TP+TN)/(TN+TP+FN+FP). b. Precision (P)=TP/(TP+FP). c. F1 score =(2*Precision (P)*Recall(R))/(Precision (P)+Recall (R)). Precision and F1 score are calculated using formulas as above, based on confusion matrix using function confusionMatrix from R package 'caret'. | | | | | |  |

**Additional file 1: eFigure 1 Precision recall curves for all models in the testing data**


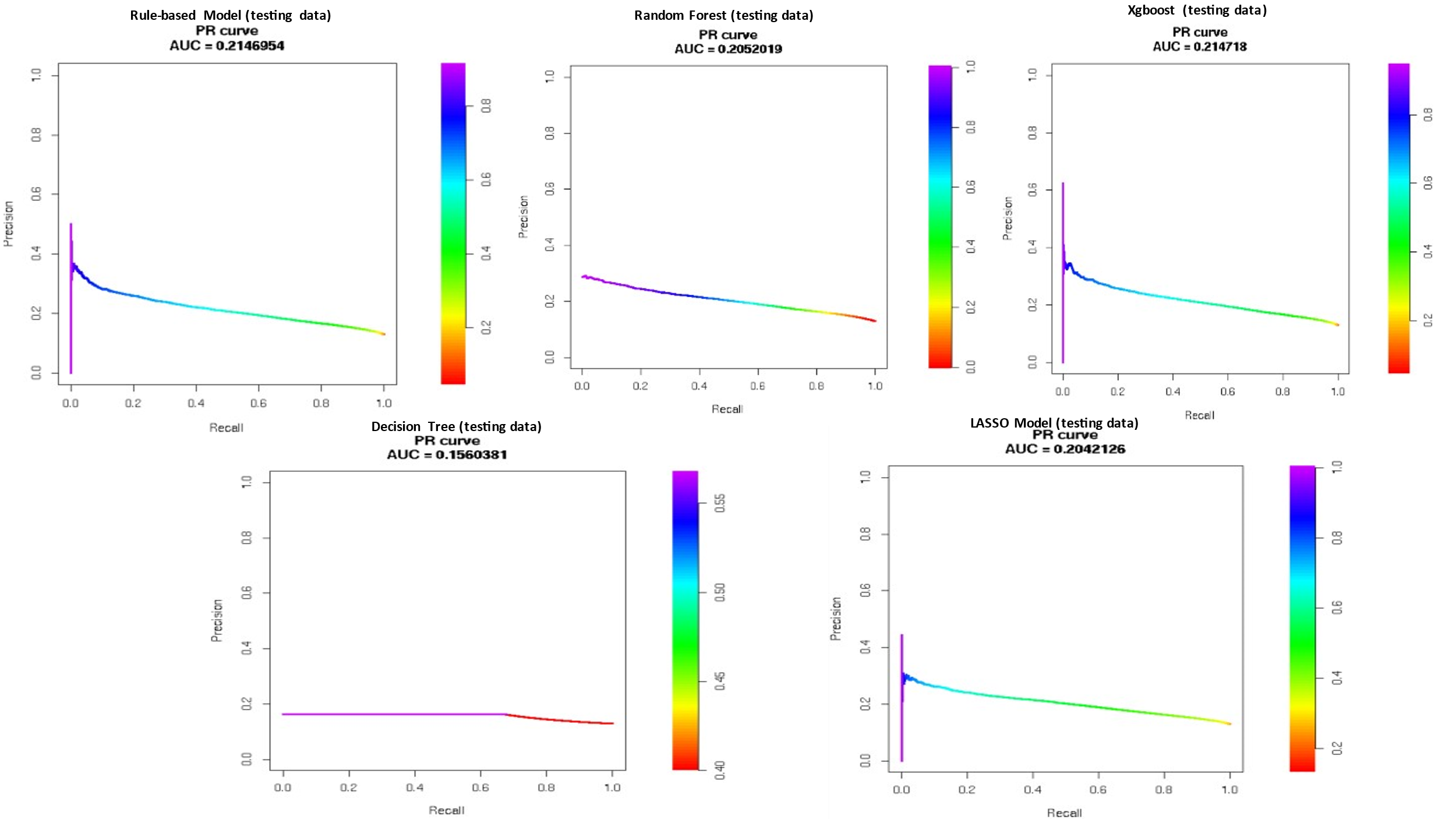


**Additional file 1: eFigure 2 Precision recall curves for all models in the training data**


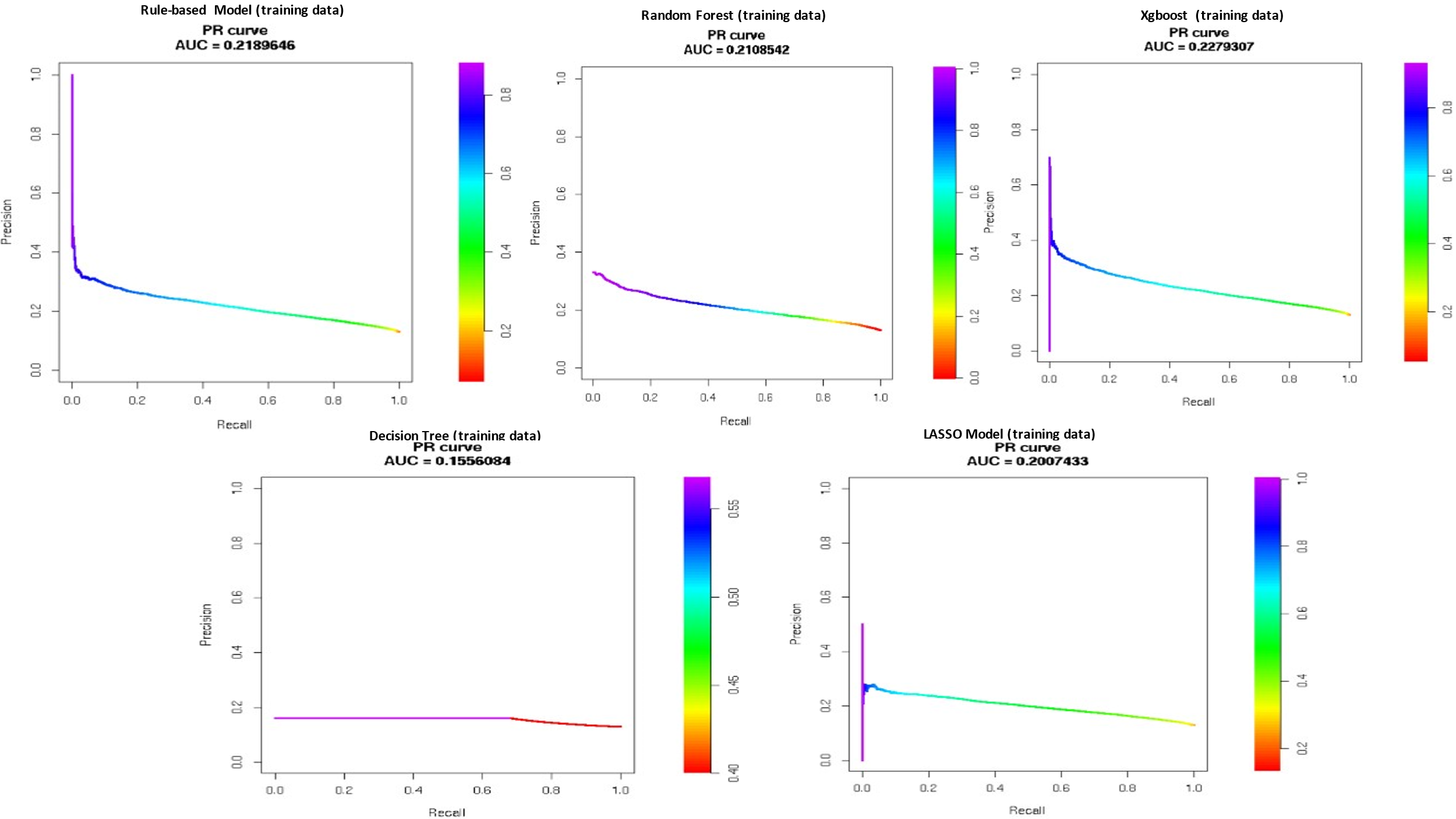

Supplement: Supplementary file 1 — Additional file 1. Model Development Parameters and Performance Metrics. [file 12911_2022_1995_MOESM1_ESM.docx]
