# Supplementary material for: Machine learning methods to predict 30-day hospital readmission outcome among US adults with pneumonia: analysis of the national readmission database
Source: BMC Med Inform Decis Mak. 2022 Nov 9;22:288. doi: 10.1186/s12911-022-01995-3 (PMC9643900; doi:10.1186/s12911-022-01995-3)
Supplement: Supplementary file 2 — Additional file 2. Study Cohort Derivation and Characteristics. [file 12911_2022_1995_MOESM2_ESM.docx]

**ADDITIONAL FILE 2**

**STUDY COHORT DERIVATION AND CHARACTERISTICS**

**eFigure 1. Study Cohort Identification**

**eTable 1. A List of All Candidate Variables From NRD Included in Study Sample**

**eTable 2. Demographic and clinical characteristics of the study sample, Listed By Overall, and readmitted Within 30 Days (Yes Vs No).**

**eTable 3. Demographic and Clinical Characteristics of the Study Sample, Listed By Overall and Training Vs Testing Cohorts.**

| **Additional file 2: eFigure 1. Study Cohort Attrition Diagram** | |
| --- | --- |
| 17,197,683 Total admissions in NRD (January 1 to Dec 31 2016) |  |
|  | Encounter-level exclusions 1,342,398 Admissions in which patient was <18 years at discharge |
| 15,855,285 Adult admissions |  |
|  | Encounter-level exclusions (5038) 79 Admission dates missing 4,960 Primary diagnosis field missing |
| 15,850,247  Eligible admissions |  |
|  | Index admission exclusions (2,335,793) 362,562 Died during admission 11,487 Survival Status missing 465,771 Same-day transfers 1,376,630 Admissions during December 276,069 Length of stay zero 989 Length of stay missing |
| 13,513,774 Eligible Index Admission |  |
|  | Index admission exclusions 13,113,764 admissions excluded due to principal diagnosis other than pneumonia |
| Eligible PND Index Admission 400,684 index admissions* 65,409 30-d readmissions** | (90,608 beyond 30-d readmission; 244,667 without any readmission) |
|  |  |
| Unique Penumonia Patient **372, 293 primary penumonia patients***** **48,280 patients with 30-d readmissions****** | (86,606 patients readmitted beyond 30 days after discharge;  237,407 patients not readmitted) |
| \| *: The records of index admissions were identified based on a list of inclusion/exclusion criteria; ** The records of 30-d readmissions were identified; *** The patient level data is identified after removing the duplicate counts of records in the same patient, and if the patient has multiple index admissions, the first index admission with 30-day readmission outcome is considered; **** Only the first index admission with 30-day readmission outcome is retained. \| \| --- \| | |

**Additional file 2: eTable 1. A List of All Candidate Variables From NRD Included in Study Sample**

| **Table 1. Overview of the Machine Learning Model Data Inputs from the 2016 NRD File** | | | |
| --- | --- | --- | --- |
| Data Category | Data Element Name | Data Description | Data Type |
| ***Demographics*** | | | |
|  | Age | Age in years at admission | Continuous |
|  | Female (Sex) | Indicator of sex | Binary |
| ***Sociodemographics*** | | | |
|  | pay1r (Payer) | Expected primary payer | Categorical |
|  | PL_NCHSr (Patient location) | NCHS urban-rural classification scheme for US counties | Categorical |
|  | zipinc_qrtlr (income quartiles) | Median household income quartiles for patient's zip code | Categorical |
| ***Medical history (co-existing) measured Elixhauser Comorbidity Measures*** | | | |
|  | AIDS | Acquired immune deficiency syndrome | Binary |
|  | ALCOHOL | Alcohol abuse |  |
|  | ARTH | Arthropathies |  |
|  | CANCER_LYMPH | Lymphoma |  |
|  | CANCER_LEUK | Leukemia |  |
|  | CANCER_METS | Metastatic cancer |  |
|  | CANCER_NSITU | Solid tumor without metastasis, in situ |  |
|  | CANCER_SOLID | Solid tumor without metastasis, malignant |  |
|  | CBVD | Cerebrovascular disease |  |
|  | CHF | Congestive heart failure |  |
|  | DEMENTIA | Dementia |  |
|  | DEPRESS | Depression |  |
|  | DIAB_UNCX | Diabetes without chronic complications |  |
|  | DIAB_CX | Diabetes with chronic complications |  |
|  | DRUG_ABUSE | Drug abuse |  |
|  | HTN_CX | Hypertension, complicated |  |
|  | HTN_UNCX | Hypertension, uncomplicated |  |
|  | LIVER_SEV | Liver disease, moderate to severe |  |
|  | LUNG_CHRONIC | Chronic pulmonary disease |  |
|  | bese | Obesity |  |
|  | PARALYSIS | Paralysis |  |
|  | PERIVASC | Peripheral vascular disease |  |
|  | RENLFL_SEV | Renal failure, severe |  |
|  | THYROID_HYPO | Hypothyroidism |  |
|  | THYROID_OTH | Other thyroid disorders |  |
|  | VALVE | Valvular disease |  |
| ***Pneumonia related conditions/medications/procedures specified by a APR_DRG (medical or surgical)*** | | | |
|  | uprespinfec | Infections of upper respiratory tract | Binary |
|  | majorchestproced | Major respiratory and chest procedures |  |
|  | otherchestproced | Other respiratory and chest procedures |  |
|  | ventilator | Respiratory system diagnosis with ventilator support 96+ hours |  |
|  | cysfpd | Cystic fibrosis-pulmonary disease |  |
|  | majorresp | Major respiratory infections and inflammations |  |
|  | bronchiolitis | Bronchiolitis and RSV pneumonia |  |
|  | otherpneu | Other pneumonia |  |
|  | hivmajor | HIV with major HIV related condition |  |
|  | extenproced | Extensive procedure unrelated to principal diagnosis |  |
|  | modproced | Moderately extensive procedure unrelated to principal diagnosis |  |
|  | nonextenproced | Nontextensive procedure unrelated to princiapl diagnosis |  |
| ***Health Use Indicators*** | | | |
|  | orproc | Indicates major (i.e., operating room) procedure found on the record | Binary |
|  | I10_NDX | Number of diagnoses on this record | Continuous |
|  | I10_NPR | Number of procedures on this record |  |
|  | I10_NECAUSE | Number of External cause codes on this record |  |
| ***Composite Severity Scores*** | | | |
|  | APRDRG_Risk_ Mortality | Risk of Mortality Subclass | Categorical |
|  | APRDRG_Severity | Severity of Illness Subclass | Categorical |
|  | mortal_score | Elixhauser Comorbidity Index | Continuous |
| ***Admission/ Discharge Information*** | | | |
|  | AWEEKEND | Admission day is a weekend | Binary |
|  | DISPUNIFORMrr | Disposition of patient | Categorical |
|  | DQTR | Discharge quarter | Categorical |
|  | ELECTIVE | Indicates elective admission | Binary |
|  | Resident | Identifies patient as a resdient of the State in which he or she received hospital care | Binary |
|  | HCUP_ED | Indicator that discharge record includes evidence of emergency department services | Categorical |
|  | LOS | Length of stay | Continuous |
| ***Hospital Information*** | | | |
|  | H_CONTRL | Control/ownership of hospital | Categorical |
|  | HOSP_BEDSIZE | Bed size of hospital |  |
|  | HOSP_UR_TEACH | Teaching status of urban hospitals |  |
|  | HOSP_URCAT4 | Hospital urban-rural designation |  |

**Additional file 2: eTable 2. Demographic and clinical characteristics of the study sample,
listed by overall and readmission vs non-readmission cohorts. (N=372,293)**

| **eTable 2. Demographic and clinical characteristics of the study sample, listed by overall and readmission vs non-readmission cohorts. (N=372,293)** | | | | |
| --- | --- | --- | --- | --- |
| **Characteristics** | **Total Overall (N= 372,293)** | **Not readmitted ithin 30 days (n=324,013)** | **Readmitted within 30 days (n=48,280)** | **P-value** |
| ***Demographics*** | | | | |
| Age, mean (SD), y | 69.07 (16.74) | 69.04 (16.78) | 69.10 (16.70) | 0.0405 |
| Women | 193,586 (52.00) | 169,599 (52.34) | 23,987 (49.68) |  |
| ***Socio-demographics*** | | | | |
| Payer | | | | |
| Medicare | 260,802 (70.05) | 224,751 (69.36) | 36,051 (74.67) | <.0001 |
| Medicaid | 38,941 (10.46) | 33,543 (10.35) | 5,398 (11.18) |  |
| Private | 54,964 (14.76) | 49,767 (15.36) | 5,197 (10.76) |  |
| Self-pay | 8,774 (2.36) | 8,080 (2.49) | 694 (1.44) |  |
| No charge | 1,102 (0.30) | 1,001 (0.31) | 101 (0.21) |  |
| Others ¹ | 7,710 (2.07) | 6,871 (2.12) | 839 (1.74) |  |
| Patient location | | | | |
| "Central" counties of metro areas of ≥1 million population | 92,605 (24.87) | 79,549 (24.55) | 13,056 (27.04) | <.0001 |
| "Fringe" counties of metro areas of ≥1 million population | 93,649 (25.15) | 81,309 (25.09) | 12,340 (25.56) |  |
| Counties in metro areas of 250,000-999,999 population | 81,282 (21.83) | 70,781 (21.85) | 10,501 (21.75) |  |
| Counties in metro areas of 50,000-249,999 population | 37,417 (10.05) | 32,793 (10.12) | 4,624 (9.58) |  |
| Micropolitan counties | 36,002 (9.67) | 31,745 (9.80) | 4,257 (8.82) |  |
| Nonmetropolitan or micropolitan counties ² | 31,338 (8.42) | 27,836 (8.59) | 3,502 (7.25) |  |
| Median household income quartile | | | | |
| Lowest income (quartile 1) ³ | 116,699 (31.35) | 101,057 (31.19) | 15,642 (32.40) | <.0001 |
| lowest to middle income (quartile 2) | 96,667 (25.97) | 84,284 (26.01) | 12,383 (25.65) |  |
| middle to high income (quartile 3) | 88,594 (23.80) | 77,252 (23.84) | 11,342 (23.49) |  |
| Highest income (quartile 4) | 70,333 (18.89) | 61,420 (18.96) | 8,913 (18.46) |  |
| Disposition | | | | |
| Routine | 207,626 (55.77) | 185,790 (57.34) | 21,836 (45.23) | <.0001 |
| Transfer to short term hospital, skilled nursing facility, intermediate care and another type of facility | 2,904 (0.78) | 2,416 (0.75) | 488 (1.01) |  |
| Other transfers | 86,595 (23.26) | 72,594 (22.40) | 14,001 (29.00) |  |
| Home health care | 70,582 (18.96) | 59,494 (18.36) | 11,088 (22.97) |  |
| Against medical advice or unknown ^4^ | 4,586 (1.23) | 3,719 (1.15) | 867 (1.80) |  |
| Resident | 358,046 (96.17) | 311,037 (96.00) | 47,009 (97.37) | <.0001 |
| ***Healthcare Use Indicators*** | | | | |
| Indicator of operating room procedure record based on ICD-10-PCS | 8,033 (2.16) | 4,032 (2.17) | 4,032 (2.15) | <.0001 |
| Number of diagnoses on this record, Mean (SD) | 13.53 (5.90) | 13.25 (5.85) | 15.41 (5.87) |  |
| Number of procedures on this record, Mean (SD) | 0.65 (1.39) | 0.61 (1.35) | 0.93 (1.64) |  |
| Number of external causes on this record, Mean (SD) | 0.18 (0.54) | 0.17 (0.53) | 0.24 (0.60) |  |
| Emergency Services Evidence | | | | |
| 0 | 42,796 (11.50) | 37,879 (11.69) | 4,917 (10.18) | <.0001 |
| 1 | 230,615 (61.94) | 200,964 (62.02) | 29,651 (61.41) |  |
| 2 | 50,677 (13.61) | 43,751 (13.50) | 6,926 (14.35) |  |
| 3 ^5^ | 48,205 (12.95) | 41,419 (12.78) | 6,786 (14.06) |  |
| ***Composite Comorbidity Scores*** | | | | |
| Severity of mortality | | | | |
| 0 | 54 (0.01) | 51 (0.02) | 3 (0.01) | <.0001 |
| 1 | 71,345 (19.16) | 66,694 (20.58) | 4,651 (9.63) |  |
| 2 | 126,242 (33.91) | 110,742 (34.18) | 15,500 (32.10) |  |
| 3 | 134,428 (36.11) | 113,301 (34.97) | 21,127 (43.76) |  |
| 4 | 40,224 (10.80) | 33,225 (10.25) | 6,999 (14.50) |  |
| Risk of illness | | | | |
| 0 | 54 (0.01) | 51 (0.02) | 3 (0.01) | <.0001 |
| 1 | 28,825 (7.74) | 27,360 (8.44) | 1,465 (3.03) |  |
| 2 | 133,142 (35.76) | 120,187 (37.09) | 12,955 (26.83) |  |
| 3 | 170,540 (45.81) | 144,442 (44.58) | 26,098 (54.06) |  |
| 4 | 39,732 (10.67) | 31,973 (9,87) | 7,759 (16.07) |  |
|  | | | | |
| ***Elixhauser Comorbidity Measures*** | | | | |
| Hypertension, uncomplicated | 170,980 (45.93) | 150,617 (46.48) | 20,363 (42.18) | <.0001 |
| Chronic Pulmonary Disease | 161,282 (43.32) | 138,562 (42.76) | 22,720 (47.06) | <.0001 |
| Hypertension, complicated | 77,643 (20.86) | 63,659 (19.65) | 13,984 (28.96) | <.0001 |
| Diabetes without chronic complications | 64,660 (17.37) | 55,722 (17.20) | 8,938 (18.51) | <.0001 |
| Hypothyroidism | 63,397 (17.03) | 54,932 (16.95) | 8,465 (17.53) | 0.0016 |
| Dementia | 53,257 (14.31) | 46,491 (14.35) | 6,766 (14.01) | 0.0502 |
| Obesity | 50,167 (13.48) | 43,625 (13.46) | 6,542 (13.55) | 0.605 |
| Depression | 51,738 (13.90) | 44,572 (13.76) | 7,166 (14.84) | <.0001 |
| Diabetes with chronic complications | 48,399 (13.00) | 40,193 (12.40) | 8,206 (17.00) | <.0001 |
| Coronary Heart Failure | 83 (0.02) | 65 (0.02) | 18 (0.04) | 0.0181 |
| AIDS | 3,417 (0.92) | 2,900 (0.90) | 517 (1.07) | 0.0002 |
| Arthropathies | 17,517 (4.71) | 15,116 (4.67) | 2,401 (4.97) | 0.0029 |
| Cerebrovascular disease | 13,301 (3.57) | 11,197 (3.46) | 2,104 (4.36) | <.0001 |
| Liver disease, moderate to severe | 670 (0.18) | 522 (0.16) | 148 (0.31) | <.0001 |
| Valvular Disease | 7,448 (2.00) | 6,285 (1.94) | 1,163 (2.41) | <.0001 |
| Peripheral Vascular Disorders | 27,569 (7.41) | 23,004 (7.10) | 4,565 (9.46) | <.0001 |
| Paralysis | 9,919 (2.66) | 8,314 (2.57) | 1,605 (3.32) | <.0001 |
| Other thyroid disorders | 4,700 (1.26) | 4,128 (1.27) | 572 (1.18) | 0.1012 |
| Renal Failure, severe | 14,339 (3.85) | 11,041 (3.41) | 3,298 (6.83) | <.0001 |
| Lymphoma | 6,333 (1.70) | 5,243 (1.62) | 1,090 (2.26) | <.0001 |
| Leukemia | 4,928 (1.32) | 4,054 (1.25) | 874 (1.81) | <.0001 |
| Metastatic Cancer | 12,372 (3.32) | 9,602 (2.96) | 2,770 (5.74) | <.0001 |
| Solid Tumor without Metastasis, in situ | 107 (0.03) | 95 (0.03) | 12 (0.02) | 0.5892 |
| Solid Tumor without Metastasis, malignant | 15,794 (4.24) | 12,820 (3.96) | 2,974 (6.16) | 0.5892 |
| Alcohol Abuse | 12,095 (3.25) | 10,144 (3.13) | 1,951 (4.04) | <.0001 |
| Drug Abuse | 11,873 (3.19) | 9,982 (3.08) | 1891 (3.92) | <.0001 |
| ***Pneumonia related conditions/medications/procedures specified by a APR_DRG (medical or surgical)*** | | | | |
| Infections of upper respiratory tract | 11,244 (3.02) | 10,325 (3.19) | 919 (1.90) | <.0001 |
| Major respiratory and chest procedures | 1,177 (0.32) | 1,020 (0.31) | 157 (0.33) | 0.7046 |
| Other respiratory and chest procedures | 1,287 (0.35) | 1,081 (0.33) | 206 (0.43) | 0.0012 |
| Respiratory system diagnosis with ventilator support 96+ hours | 2,470 (0.66) | 1,977 (0.61) | 493 (1.02) | <.0001 |
| Cystic fibrosis-pulmonary disease | 580 (0.16) | 506 (0.16) | 74 (0.15) | 0.8804 |
| Major respiratory infections and inflammations | 74,040 (19.89) | 62,933 (19.42) | 11,107 (23.01) | <.0001 |
| Bronchiolitis and RSV pneumonia | 1,068 (0.29) | 959 (0.30) | 109 (0.23) | 0.0071 |
| Other pneumonia | 272,680 (73.24) | 238,901 (73.73) | 33,779 (69.96) | <.0001 |
| HIV with major HIV related condition | 599 (0.16) | 498 (0.15) | 101 (0.21) | 0.0045 |
| Extensive procedure unrelated to principal diagnosis | 849 (0.23) | 660 (0.20) | 189 (0.39) | <.0001 |
| Moderately extensive procedure unrelated to principal diagnosis | 2,784 (0.75) | 2,242 (0.69) | 542 (1.12) | <.0001 |
| Nonextensive procedure unrelated to principal diagnosis | 1,223 (0.33) | 958 (0.30) | 265 (0.55) | <.0001 |
| ***Admission/Discharge*** | | | | |
| Weekend Admission | 95,744 (25.72) | 83,538 (25.78) | 12,206 (25.28) | 0.0189 |
| Discharge Quarter | | | | |
| Jan-Mar | 136,026 (36.54) | 119,345 (36.83) | 16,681 (34.55) | <.0001 |
| Apr-Jun | 102,645 (27.57) | 89,536 (27.63) | 13,109 (27.15) |  |
| Jul-Sep | 79,686 (21.40) | 68,810 (21.24) | 10,876 (22.53) |  |
| Oct-Dec | 53,936 (14.49) | 46,322 (14.30) | 7,614 (15.77) |  |
| Elective Admission | | | | |
| Yes | 15,257 (4.10) | 13,656 (4.21) | 1,601 (3.32) | <.0001 |
| No | 356,745 (95.82) | 310,088 (95.70) | 46,657 (96.64) |  |
| Unknown ^5^ | 291 (0.08) | 269 (0.08) | 22 (0.05) |  |
| Length of Stay, Mean/SD | 5.00 (4.75) | 4.83 (4.56) | 6.10 (5.74) | <.0001 |
| ***Hospital Information*** | | | | |
| Hospital Ownership | | | | |
| Government | 44,545 (11.97) | 38,977 (12.03) | 5,568 (11.53) | <.0001 |
| Private, not-for-profit | 268,778 (72.20) | 233,956 (72.21) | 34,822 (72.13) |  |
| Private, investor-owned | 268,778 (72.20) | 51,080 (15.76) | 7,890 (16.34) |  |
| Hospital Bedsize | | | | |
| Small | 74,748 (20.08) | 66,051 (20.39) | 8,697 (18.01) | <.0001 |
| Medium | 113,504 (30.49) | 98,956 (30.54) | 14,548 (30.13) |  |
| Large | 184,041 (49.43) | 159,006 (49.07) | 25,035 (51.85) |  |
| Hospital teaching status | | | | |
| Metropolitan non-teaching | 124,707 (33.50) | 108,559 (33.50) | 16,148 (33.45) | <.0001 |
| Metropolitan teaching | 195,631 (52.55) | 169,077 (52.18) | 26,554 (55.00) |  |
| Non-metropolitan | 51,955 (13.96) | 46,377 (14.31) | 5,578 (11.55) |  |
| Hospital urban-rural designation | | | | |
| Large metropolitan areas with at least 1 million residents | 191,776 (51.51) | 165,488 (51.07) | 26,288 (54.45) | <.0001 |
| Small metropolitan areas with less than 1 million residents | 128,562 (34.53) | 112,148 (34.61) | 16,414 (34.00) |  |
| Micropolitan areas | 33,645 (9.04) | 29,801 (9.20) | 3,844 (7.96) |  |
| Not metropolitan or micropolitan | 18,310 (4.92) | 16,576 (5.12) | 1,734 (3.59) |  |
| 1. Includes payer of other type or missing; 2. Includes patient locations of other type or missing; 3. Includes the median household income category of lowest income (income quartiles = 1) or missing; 4: Includes destination of other type, unknown or missing; 5. Includes elective status of other type, unknown or missing. | | | | |

**Additional file 2: eTable 3. Demographic and Clinical Characteristics of the Study Sample, Listed By Overall and Training Vs Testing Cohorts**

| **eTable 3. Demographic and clinical characteristics of the study sample, listed by overall and training vs testing cohorts. (N=372,293)** | | | | |
| --- | --- | --- | --- | --- |
| **Characteristics** | Total Overall (N=372,293) | Training (n=186,147) | Testing (n=186,146) | P-value |
| ***Demographics*** | | | | |
| **Age, mean (SD), y** | 69.07 (16.74) | 69.04 (16.78) | 69.10 (16.70) | 0.0405 |
| Women | 193,586 (52.00) | 96,962 (52.09) | 96,624 (51.91) | 0.2668 |
| ***Socio-demographics*** | | | | |
| Payer | | | | |
| Medicare | 260,802 (70.05) | 130,277 (69.99) | 130,525 (70.12) | 0.3347 |
| Medicaid | 38,941 (10.46) | 19,585 (10.52) | 19,356 (10.40) |  |
| Private | 54,964 (14.76) | 27,427 (14.73) | 27,537 (14.79) |  |
| Self-pay | 8,774 (2.36) | 4,362 (2.34) | 4,412 (2.37) |  |
| No charge | 1,102 (0.30) | 565 (0.30) | 537 (0.29) |  |
| Others ^1^ | 7,710 (2.07) | 3,930 (2.11) | 3,780 (2.03) |  |
| Patient location | | | | |
| "Central" counties of metro areas of ≥1 million population | 92,605 (24.87) | 46,391 (24.92) | 46,214 (24.83) | 0.9739 |
| "Fringe" counties of metro areas of ≥1 million population | 93,649 (25.15) | 46,834 (25.16) | 46,815 (25.15) |  |
| Counties in metro areas of 250,000-999,999 population | 81,282 (21.83) | 40,648 (21.84) | 40,634 (21.83) |  |
| Counties in metro areas of 50,000-249,999 population | 37,417 (10.05) | 18,678 (10.03) | 18,739 (10.07) |  |
| Micropolitan counties | 36,002 (9.67) | 17,978 (9.66) | 18,024 (9.68) |  |
| Non metropolitan or micropolitan counties^2^ | 31,338 (8.42) | 15,617 (8.39) | 15,721 (8.45) |  |
| Median household income quartile | | | | |
| Lowest income (quartile 1)^3^ | 116,699 (31.35) | 58,399 (31.37) | 58,300 (31.32) | 0.9766 |
| Lowest to middle income (quartile 2) | 96,667 (25.97) | 48,284 (25.94) | 48,383 (25.99) |  |
| Middle to high income (quartile 3) | 88,594 (23.80) | 44,311 (23.80) | 44,283 (23.79) |  |
| Highest income (quartile 4) | 70,333 (18.89) | 35,152 (18.88) | 35,181 (18.90) |  |
| Disposition | | | | |
| Routine | 207,626 (55.77) | 103,856 (55.79) | 103,770 (55.75) | 0.4757 |
| Transfer to short term hospital, skilled nursing facility, itermediate care and another type of facility | 2,904 (0.78) | 1,485 (0.80) | 1,419 (0.76) |  |
| Other transfers | 86,595 (23.26) | 43,388 (23.31) | 43,207 (23.21) |  |
| Home health care | 70,582 (18.96) | 35,123 (18.87) | 35,459 (19.05) |  |
| Against medical advice or unknown ^4^ | 4,586 (1.23) | 2,294 (1.23) | 2,292 (1.23) |  |
| Resident | 358,046 (96.17) | 179,036 (96.18) | 179,010 (96.17) | 0.8178 |
| ***Admission/Discharge*** | | | | |
| Weekend Admission | 95,744  25.72 | 47,916 (25.74) | 47,828 (25.69) | 0.7407 |
| Discharge Quarter | | | | |
| Jan-Mar | 136,026 (36.54) | 67,967 (36.51) | 68,059 (36.56) | 0.3895 |
| Apr-Jun | 102,645 (27.57) | 51,548 (27.69) | 51,097 (27.45) |  |
| Jul-Sep | 79,686 (21.40) | 39,737 (21.35) | 39,949 (21.46) |  |
| Oct-Dec | 53,936 (14.49) | 26,894 (14.45) | 27,042 (14.53) |  |
| Elective Admission | | | | |
| Yes | 15,257 (4.10) | 7,740 (4.16) | 7,517 (4.04) | 0.1355 |
| No | 356,745 (95.82) | 178,254 (95.76) | 178,491 (95.89) |  |
| Unknown | 291 (0.08) | 152 (0.08) | 139 (0.07) |  |
| Length of Stay (mean/SD), day |  | 5.01 (4.81) | 4.99 (4.69) | 0.0385 |
| ***Healthcare Use Indicators*** | | | | |
| Number of diagnoses on this record, Mean (SD) | 13.53 (5.90) | 13.54 (5.91) | 13.53 (5.89) | 0.9239 |
| Number of procedures on this record, Mean (SD) | 0.65 (1.39) | 0.66 (1.41) | 0.65 (1.38) | 0.0076 |
| Number of external causes on this record, Mean (SD) | 0.18 (0.54) | 0.18 (0.53) | 0.18 (0.54) | 0.9956 |
| Emergency Services Evidence | | | | |
| 0 | 42,796 (11.50) | 21,416 (11.50) | 21,380 (11.49) | 0.37 |
| 1 | 230,615 (61.94) | 115,064 (61.81) | 115,551 (62.08) |  |
| 2 | 50,677 (13.61) | 25,469 (13.68) | 25,208 (13.54) |  |
| *3 ^5^* | 48,205 (12.95) | 24,197 (13.00) | 24,008 (12.90) |  |
| Indicator of operating room procedure record based on ICD-10-PCS | 8,033 (2.16) | 4,001 (2.15) | 4,032 (2.17) | 0.7268 |
| ***Elixhauser Comorbidity Measures*** | | | | |
| Hypertension, uncomplicated | 170,980 (45.93) | 85,281 (45.81) | 85,699 (46.04) | 0.1697 |
| Hypertension, complicated | 77,643 (20.86) | 38,798 (20.84) | 38,845 (20.87) | 0.8503 |
| Diabetes without chronic complications | 64,660 (17.37) | 32,427 (17.42) | 32,233 (17.32) | 0.4009 |
| Hypothyroidism | 63,397 (17.03) | 31,746 (17.05) | 31,651 (17.00) | 0.6782 |
| Dementia | 53,257 (14.31) | 26,393 (14.18) | 26,864 (14.43) | 0.0275 |
| Obesity | 50,167 (13.48) | 25,400 (13.65) | 24,767 (13.31) | 0.0024 |
| Depression | 51,738 (13.90) | 25,879 (13.90) | 25,859 (13.89) | 0.924 |
| Diabetes with chronic complications | 48,399 (13.00) | 24,141 (12.97) | 24,258 (13.03) | 0.569 |
| Coronary Heart Failure | 83 (0.02) | 44 (0.02) | 39 (0.02) | 0.5831 |
| AIDS | 3,417 (0.92) | 1,739 (0.93) | 1,678 (0.90) | 0.2944 |
| Arthropathies | 17,517 (4.71) | 8,710 (4.68) | 8,807 (4.73) | 0.453 |
| Cerebrovascular disease | 13,301 (3.57) | 6,688 (3.59) | 6,613 (3.55) | 0.5076 |
| Liver disease, moderate to severe | 670 (0.18) | 336 (0.18) | 334 (0.18) | 0.9383 |
| Valvular Disease | 7,448 (2.00) | 3,700 (1.99) | 3,748 (2.01) | 0.5744 |
| Peripheral Vascular Disorders | 27,569 (7.41) | 13,646 (7.33) | 13,923 (7.48) | 0.0831 |
| Paralysis | 9,919 (2.66) | 5,026 (2.70) | 4,893 (2.63) | 0.1758 |
| Other thyroid disorders | 4,700 (1.26) | 2,398 (1.29) | 2,302 (1.24) | 0.1587 |
| Renal Failure, severe | 14,339 (3.85) | 7,276 (3.91) | 7,063 (3.79) | 0.0696 |
| Lymphoma | 6,333 (1.70) | 3,178 (1.71) | 3,155 (1.69) | 0.7705 |
| Leukemia | 4,928 (1.32) | 2,507 (1.35) | 2,421 (1.30) | 0.2174 |
| Metastatic Cancer | 12,372 (3.32) | 6,260 (3.36) | 6,112 (3.28) | 0.1759 |
| Solid Tumor without Metastasis, in situ | 107 (0.03) | 53 (0.03) | 54 (0.03) | 0.923 |
| Solid Tumor without Metastasis, malignant | 15,794 (4.24) | 7,918 (4..25) | 7,876 (4.23) | 0.7325 |
| Alcohol Abuse | 12,095 (3.25) | 6,080 (3.27) | 6,015 (3.23) | 0.5477 |
| Chronic Pulmonary Disease | 161,282 (43.32) | 80,585 (43.29) | 80,697 (43.35) | 0.7121 |
| Drug Abuse | 11,873 (3.19) | 5,982 (3.21) | 5,891 (3.16) | 0.3958 |
| ***Pneumonia related conditions/medications/procedures specified by a APR_DRG (medical or surgical)*** | | | | |
| Infections of upper respiratory tract | 11,244 (3.02) | 5,634 (3.03) | 5,610 (3.01) | 0.818 |
| Major respiratory and chest procedures | 1,177 (0.32) | 575 (0.31) | 602 (0.32) | 0.4306 |
| Other respiratory and chest procedures | 1,287 (0.35) | 677 (0.36) | 610 (0.33) | 0.0614 |
| Respiratory system diagnosis with ventilator support 96+ hours | 2,470 (0.66) | 1,215 (0.65) | 1,255 (0.67) | 0.4194 |
| Cystic fibrosis-pulmonary disease | 580 (0.16) | 270 (0.15) | 310 (0.17) | 0.0965 |
| Major respiratory infections and inflammations | 74,040 (19.89) | 36,981 (19.87) | 37,059 (19.91) | 0.7494 |
| Bronchiolitis and RSV pneumonia | 1,068 (0.29) | 539 (0.29) | 539 (0.28) | 0.7592 |
| Other pneumonia | 272,680 (73.24) | 136,351 (73.25) | 136,329 (73.24) | 0.9329 |
| HIV with major HIV related condition | 599 (0.16) | 319 (0.17) | 280 (0.15) | 0.1107 |
| Extensive procedure unrelated to principal diagnosis | 849 (0.23) | 426 (0.23) | 423 (0.23) | 0.9178 |
| Moderately extensive procedure unrelated to principal diagnosis | 2,784 (0.75) | 1,394 (0.75) | 1,390 (0.75) | 0.9392 |
| Nontextensive procedure unrelated to princiapl diagnosis | 1,223 (0.33) | 569 (0.31) | 654 (0.35) | 0.0149 |
| ***Composite Severity Scores*** | | | | |
| Severity of mortality | | | | |
| 0 | 54 (0.01) | 22 (0.01) | 32 (0.02) | 0.2687 |
| 1 | 71,345 (19.16) | 35,804 (19.23) | 35,541 (19.09) |  |
| 2 | 126,242 (33.91) | 63,087 (33.89) | 63,155 (33.93) |  |
| 3 | 134,428 (36.11) | 67,014 (36.00) | 67,414 (36.22) |  |
| 4 | 40,224 (10.80) | 20,219 (10.86) | 20,005 (10.75) |  |
| Risk of illness | | | | |
| 0 | 54 (0.01) | 22 (0.01) | 32 (0.02) | 0.4256 |
| 1 | 28,825 (7.74) | 14,412 (7.74) | 14,413 (7.74) |  |
| 2 | 133,142 (35.76) | 66,733 (35.85) | 66,409 (35.68) |  |
| 3 | 170,540 (45.81) | 85,065 (45.70) | 85,475 (45.92) |  |
| 4 | 39,732 (10.67) | 19,914 (10.70) | 19,818 (10.65) |  |
| Elixhauser Comorbidity Index Scores, Mean (SD) | 0.54 (4.74) | 0.5360 (4.7542) | 0.5373 (4.7328) | 0.9352 |
| ***Hospital Information*** | | | | |
| Hospital Ownership | | | | |
| Government | 44,545 (11.97) | 22,494 (12.08) | 22,051 (11.85) | 0.0585 |
| Private, not-for-profit | 268,778 (72.20) | 134,117 (72.05)_ | 134,661 (72.34) |  |
| Private, investor-owned | 58,970 (15.84) | 29,535 (15.87) | 29,435 (15.81) |  |
| Hospital Bedsize | | | | |
| Small | 74,748 (20.08) | 37,522 (20.16) | 37,226 (20.00) | 0.4464 |
| Medium | 113,504 (30.49) | 56,645 (30.43) | 56,859 (30.55) |  |
| Large | 184041 (49.43) | 91979 (49.41) | 92062 (49.46) |  |
| Hospital teaching status | | | | |
| Meropolitan non-teaching | 124,707 (33.50) | 62,360 (33.50) | 62,347 (33.49) | 0.9474 |
| Meropolitan teaching | 195,631 (52.55) | 97,777 (52.53) | 97,854 (52.57) |  |
| Non-metropolitan | 51,955 (13.96) | 26,009 (13.97) | 25,946 (13.94) |  |
| Hospital urban-rural designation | | | | |
| Large metropolitan areas with at least 1 million residents | 191,776 (51.51) | 96,058 (51.60) | 95,718 (51.42) | 0.3736 |
| Small metropolitan areas with less than 1 million residents | 128,562 (34.53) | 64,079 (34.42) | 64,483 (34.64) |  |
| Micropolitan areas | 33,645 (9.04) | 16,784 (9.02) | 16,861 (9.06) |  |
| Not metropolitan or micropolitan | 18,310 (4.92) | 9,225 (4.96) | 9,085 (4.88) |  |
| 1. Includes payer of other type or missing; 2. Includes patient locations of other type or missing; 3. Includes the median household income category of lowest income (income quartiles = 1) or missing; 4: Includes destination of other type, unknown or missing; 5. Includes elective status of other type, unknown or missing. | | | | |
